# PROTOCOL: The effects of agricultural output market access interventions on agricultural, socio‐economic and food and nutrition security outcomes in low‐ and middle‐income countries: A systematic review

**DOI:** 10.1002/cl2.1348

**Published:** 2023-08-21

**Authors:** Paul Fenton Villar, Tomasz Kozakiewicz, Vinitha Bachina, Sarah Young, Shannon Shisler

**Affiliations:** ^1^ School of International Development University of East Anglia Norwich UK; ^2^ International Initiative for Impact Evaluation (3ie) Washington District of Columbia USA; ^3^ CMU Libraries Carnegie Mellon University Pittsburgh Pennsylvania USA

## Abstract

Development agencies and international donors’ efforts are increasingly focusing on better integrating poor and remote farmers into agricultural markets to address the chronic issues of rural poverty and hunger in low‐ and middle‐income countries. Using systematic methods for information retrieval, critical appraisal and evidence synthesis, this research aims to examine evidence on the effects of five focal types of agricultural market access interventions: (i) farm‐to‐market transport infrastructure interventions; (ii) output market information interventions; (iii) initiatives creating new marketplaces and alternative marketing opportunities; (iv) contract farming initiatives; (v) interventions improving storage infrastructure. In this review, we will study evidence of the magnitude and direction of intervention effects on agricultural, socio‐economic, and food and nutrition security outcomes. We will examine evidence of the distribution of reported effects across different contexts, interventions and sub‐groups of the population (e.g., according to sex, socio‐economic status, farm size, etc.). We will also report on included studies’ risk of bias and on what evidence is available on intervention costs, or their cost‐effectiveness. This protocol outlines this review's planned methods and the criteria for selecting and including studies in its analysis.

## 1 BACKGROUND

### 1.1 The problem, condition or issue

Despite progress in recent decades, hunger and rural poverty remain chronic global development challenges. In 2021, more than 698 million people were living in poverty and 828 million people were chronically food insecure (FAO et al., 2022; Suckling et al., 2021). Estimates indicate that approximately 80% of the world's poor live in rural areas and that the livelihoods of almost half of the world's undernourished people and 63 per cent of those in poverty are dependent on farming, particularly small‐scale and subsistence farming (Fan & Rue, 2020; World Bank, 2020, 2022).

Today, a key aspect of development agencies and international donors’ efforts to address rural poverty and hunger includes better integrating rural farmers into agricultural markets and promoting a shift from subsistence to market‐oriented agriculture (IFAD, 2022; World Bank, 2022). For example, following the 2008 World Development Report on Agriculture (World Bank, 2007), the World Bank developed two successive Agriculture Action Plans (FY10‐12 and FY13‐15) that identified linking farmers to markets as one of five priority areas necessary to support the development of the agricultural sector and alleviate poverty. In 2015, it also identified improving farmer market access as one of three key actions in its Agenda for the Global Food System, which was prepared as a call for action to provide support for the Sustainable Development Goals on ending hunger and poverty (World Bank Group, 2015). Similarly, in another example, the Food Coalition program^1^ describes improving access and participation in markets as an important long‐term solution to transforming global agrifood systems (FAO, 2022).

The underlying premise supporting the promotion of increased market participation traces its origins back to, at least, Adam Smith and David Ricardo: who highlighted the welfare gains that can be achieved from choosing market‐oriented production and exchange compared to operating under autarkic conditions (Barrett, 2008). More modern arguments focusing specifically on the agricultural sector further highlight that it is anticipated improving market participation will incentivise farmers to cultivate higher‐value crops (including cash crops), adopt improved inputs and increase their agricultural production. In turn, it is expected that increased market sales and agricultural production will also help to increase farmers’ income and support their food security by improving food access and affordability (Barrett, 2008; Barrett et al., 2010; Chamberlin & Jayne, 2013; Gómez et al., 2011; de Janvry & Sadoulet, 2020).

However, data indicates farmers’ participation in markets remains limited. Research shows that approximately 35% of farmers worldwide are producing food as part of formal or informal supply chains (Farmer Income Lab, 2018). Furthermore, there are numerous factors inhibiting farmers’ access and participation in output markets. Constraints caused by physical access to markets (including time and distance) have been a principal focus of the literature on farmers’ market access (Baltenweck & Staal, 2007). Other highlighted factors related to farmers’ limited market access concern farmers’ lack of connections to established market actors, land rights, market risk, market structure, low bargaining power, low economies of scale, barriers to entry, anti‐competitive behaviour by market actors, economic and trade policies, and a lack of capital (human, social, financial, natural) (Aquino, 1998; Chamberlin & Jayne, 2013; de Brauw & Bulte, 2021; Maitre et al., 2011; Markelova et al., 2009; Maziku & Mashenene, 2020; Platteau, 1996).

Several discussions have also focused on the costs of accessing markets (e.g., including information and coordination costs). These costs can drive an effective wedge between input and output prices, rendering market participation non‐remunerative. Similarly, receipt of low farm gate prices can render market participation non‐remunerative (Alene et al., 2008; Barrett, 2008; de Brauw & Bulte, 2021; de Janvry et al., 1991). Price volatility across area and time and unpredictability also lead to uncertainty and exposes farmers to significant risk (Cardell & Michelson, 2020).

### 1.2 The intervention

In this review, we will compile evidence on the effects of a selection of five key types of interventions intended to improve farmers’ market access and participation in low‐ and middle‐income countries (LMICs). Informed by funder demand and expert suggestions, we examine interventions that address farmers’ access and participation in output markets by (i) improving farm‐to‐market transport infrastructure; (ii) increasing access to output market information; (iii) creating new marketplaces or alternative marketing opportunities; (iv) facilitating contract farming; (v) improving storage infrastructure.

#### 1.2.1 Farm to market transport infrastructure

Transporting produce to markets can be costly if farmers must rely on slow, poor quality transport networks and if trade, therefore, involves the risk of the loss of product quality and spoilage. Chamberlin and Jayne (2013) show rural marketing costs in many LMICs are often dominated by transportation costs and de Brauw and Bulte (2021) explain that such transaction costs reduce the benefits and incentives to trade. Farm to market transport infrastructure interventions include initiatives that improve infrastructure, such as road and bridges, used for delivering agricultural produce to domestic and international markets. This includes initiatives constructing, rehabilitating, or maintaining infrastructure. They are intended to reduce the risks and costs associated with transporting goods and produce. This may make market opportunities more lucrative and improve access to new or distant markets (Aggarwal et al., 2022; Ludwig et al., 2016).

#### 1.2.2 Output market information

Output market information interventions include two different types of information interventions. One class of interventions include initiatives that directly provide farmers with information about the market, usually about prices, completed trades, or current market demand (including information on location, quality, variety, and quantity demanded). This can help inform farmers about markets where they might sell their produce, as well as help them to decide what to grow and how much to expect to sell it for. A second class of interventions increase access to technologies required to access market information. This includes investments in mobile phone services, internet or broadband access, and the basic provision of electricity.

Since Stigler's seminal work on the ‘Economics of Information’ (Stigler, 1961), a large body of literature has shown asymmetric information can affect market equilibria, creating an inefficient allocation of goods across markets, increasing price dispersion and instability, and decreasing trade and competition (Aker, 2008; Goyal, 2010). For example, one way information asymmetries can distort agriculture markets is that more informed traders may use their knowledge to exploit farmers and pay lower farm gate prices. Farmers who receive lower prices for their produce may be less inclined to trade and may limit their market operations accordingly (Magesa et al., 2020; Nakasone, 2013).^2^ Overall, it is anticipated market information may facilitate deeper agricultural markets by both encouraging and enabling farmers to participate more effectively in them (Giovannucci & Shepherd, 2007).

#### 1.2.3 New marketplaces and alternative marketing opportunities

Interventions creating new marketplaces and alternative marketing opportunities aim to provide new ways for market participants to trade and/or reduce search costs by simplifying or improving connections between market participants. It is expected that these types of interventions will increase market competition, improve the prices farmers receive for their produce and, to the extent this creates market incentives, increase production and possibly alter the production of the types of crops grown (e.g., increasing the adoption of cash crops) (Goyal, 2010; Levi et al., 2020). Examples of this type of intervention include creating new marketplace by connecting geographically distributed agri‐markets through online platforms (e.g., see Levi et al., 2020) or mobile trading platforms (e.g., see Bergquist & McIntosh, 2021). Other examples of alternative marketing outlets include setting up trading hubs or internet kiosks (e.g., Goyal, 2010) or curating commitments or stable arrangements where traders offer to buy farmers’ produce (e.g., Bold et al., 2022; Maertens et al., 2020).

#### 1.2.4 Contract farming

Contract farming and outgrower schemes create arrangements where traders and farmers enter forward‐looking agreements for the delivery of agricultural produce (Eaton & Shepherd, 2001).^3^ As well as advanced knowledge of price, these arrangements can provide farmers with more certainty about the quantity and quality of produce in demand. Contracts can reduce some of the risks associated with farmers’ land allocation and investment decisions and they can limit the need to use spot markets to sell produce after they have already invested in the inputs required to farm their crops (Bellemare & Bloem, 2018; Bellemare & Lim, 2018). Increased certainty regarding price and demand can also facilitate farmers’ investment into new markets, as well as access to finance (e.g., via credit facilities or sometimes the conditions of a contract can provide inputs in‐kind). For example, in the contracts studied by Bellemare (2010), seeds, pesticides, and fertilizer are provided to farmers with the contracted crop being used as collateral. It may also help farmers organise supply under a common contractual agreement, thereby accessing new markets that require or benefit from some degree of aggregation. Where aggregation improves farmers’ negotiation or bargaining power, this could also result in improved terms of trade and increase the prices they receive for their produce (Barrett et al., 2012).

#### 1.2.5 Storage infrastructure

Improved storage infrastructure interventions provide access to storage facilities, such as sheds with off‐the‐ground storage, and storage deposit systems. These interventions intend to reduce the risks associated with the storage of agricultural produce, which may help to increase the cultivation of alternative high‐yielding crops that do not otherwise store well. This can increase access to new or alternative markets (Kantunze et al., 2017; Omotilewa et al., 2018) and it may also improve market access by enabling farmers to perform a more extensive market search for buyers or enabling them to wait for more favourable market conditions (i.e., perform intertemporal arbitrage) (Lothoré & Delmas, 2009; Pingali et al., 2019). However, an increasing degree of controversy now surrounds storage interventions. For example, Cardell and Michelson (2020) show that the price for maize often declines after harvest (~30% of the time), so storing after harvest may also be detrimental to farmers outcomes (especially when programs are combined with credit facilities).

### 1.3 How the intervention might work

**Figure 1:**
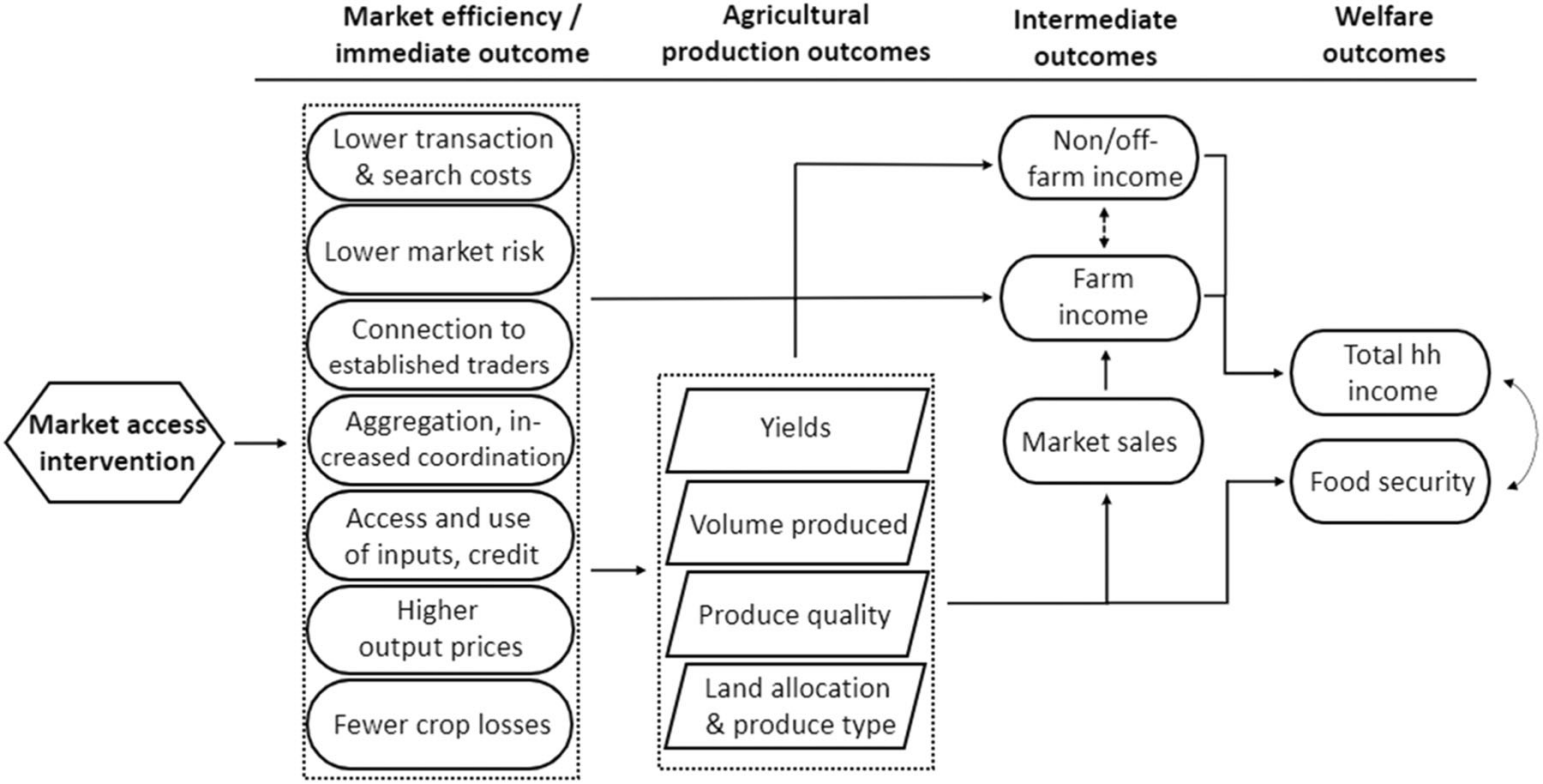
Conceptual model: Potential channels of effects.

The theoretical framework for this review is based on a broader literature that indicates interventions can strengthen market access by affecting the way markets function and minimising the market inefficiencies that inhibit farmers market access and participation (de Castro, 2021). This includes, for instance, improving connectivity or decreasing farmers’ search and transaction costs, lowering farmers’ market risk, increasing the prices farmers receive for their produce, and so forth (see the implied conceptual model in Figure 1).

Both theory and evidence suggest that by improving factors which increase the prices farmers receive for their produce, decrease market risks, and reducing search and transaction costs, output market access interventions can affect production outcomes. This occurs as the returns to selling produce in the market (commercialisation) increases relative to the welfare benefits of growing staple crops for consumption. Alternatively, this may also incentivize farmers to expand their cultivation of crops and other agricultural products (Bold et al., 2022; de Brauw & Bulte, 2021; Goyal, 2010).

To the extent that this causes farmers to adjust their decisions regarding their land allocation, this may also cause an increase in the adoption of alternative high‐value crops and increase the production of higher quality crops (Bold et al., 2022). For example, work by Fafchamps (1992) indicates that price risk affects farmers’ crop choice. Similarly, to the extent these factors enable greater access to credit and market incentives encourage the use of improved inputs, it is anticipated this may increase agriculture yields (George, 2014; Ragasa et al., 2018).

Increased commercialisation, higher returns to trade, and larger yields are expected to increase farmers’ incomes (Barrett, 2008; Zeller et al., 1998). Haggblade and colleagues (1989), Haggblade and Hazell (1989) and de Janvry and Sadoulet (2002) also explain it is possible that agricultural growth can generate income and employment multipliers in the rural non‐farm economy by increasing demand for production inputs and consumer goods. This may further increase farmers off‐farm income and where production outcomes, yields and incomes increase, it is anticipated interventions will help to support farmers’ food and nutrition security by improving food access or whether they can afford to purchase food (Barrett et al., 2010; Barrett, 2008; Mishra et al., 2018; Rugumamu, 2014).

However, there are also several competing predictions about the anticipated effects of market access interventions. For instance, farmers’ incomes can be diversified on‐farm but also off‐farm (Reardon, 1998). Brooks and Donovan (2020) highlight that households manage their choice of agricultural production and labour market risk simultaneously, and that farm investment is risky. This nuance creates a degree of theoretical ambiguity about the potential effects of these interventions on farmers’ agricultural outcomes. If market access interventions, such as those improving roads and other forms of transport, simultaneously alter farmers’ access to other income‐generating opportunities, then these interventions may not increase farm incomes as implied above. The anticipated effects on farm incomes become ambiguous because households may substitute some of their farm activities with new opportunities that arise off‐farm, for example, in the labour market.

Furthermore, where market access interventions cause a shift away from staple crops and towards higher‐value cash crops, they may have an ambiguous effect on food security. Theoretically, if the price of the cash crop were to fall, the farmers’ income from cash crops may not be capable of fulfilling their dietary needs. An extensive and longstanding debate concerns such issues (e.g., see Alarcon, 1993; Barbier, 1989; Bouis & Haddad, 1990; Dewey, 1981; Fafchamps, 1992; Govereh & Jayne, 2003; Timmer, 1997; etc.).

Increasing market access may also lead to higher market and price competition (Minot & Hill, 2007). For example, transport infrastructure may also bring more goods to local markets to compete with local farmers, thereby possibly decreasing prices and local farmers’ incomes (Dumas & Játiva, 2020). A growing literature also concerns competition and market structure (e.g., see Bergquist & Dinerstein, 2020; Casaburi & Reed, 2021; Chatterjee, 2019). If intermediaries are operating in a competitive environment, then market access interventions that reduce transaction costs would also yield savings that traders pass on to farmers (in the form of higher prices). However, if traders are exercising a high degree of market power, cost savings may almost exclusively benefit (be captured by) intermediaries, not farmers (see Bergquist & Dinerstein, 2020).

Finally, market participation can introduce market and commercial risks that can be difficult to manage (particularly when farmers are highly leveraged) and it can also increase farmers’ costs. For example, market price risks are already well documented above. Furthermore, contract farming can introduce risks, such as risk of contractual default, while also creating additional costs (e.g., due to the inputs requirements for special varieties and to manage the processes necessary to fulfill a contract) (Narayanan, 2014). This emphasises the risk‐return trade‐off farmers may face when participating in markets.

Across all these interventions, the extent of the positive and unintended negative effects described above are likely to vary considerably across farmers’ characteristics and over time. A reduction in transaction costs will affect farmers differently based on the type and level of sales. Also, it is reasonable to assume that farmers face non‐separable production and consumption decisions, due to market failures and missing markets, meaning that consumption decisions would influence production decision and vice‐versa (Key et al., 2000; Taylor & Adelman, 2003). Farmers can be distinguished into two groups: net‐buying farmers (including non‐agricultural households and subsistence farmers) and net‐selling farmers. Farmers’ production and consumption choices vary from farmer to farmer. Similarly, effects in the short term are not necessarily the same as those in the long‐term (Lane et al., in press).

### 1.4 Why it is important to do this review

It is widely argued that market access may be a necessary (if not sufficient) condition required for agricultural transformation and poverty reduction (Barrett et al., 2010; Barrett, 2008; Chamberlin & Jayne, 2013; Gómez et al., 2011; de Janvry & Sadoulet, 2020). Reflecting these arguments, development agencies and international donors’ efforts to address poverty and rural hunger increasingly focus on better integrating poor and remote farmers into markets. For example, the International Fund for Agricultural Development (IFAD) (2022) states increasing poor rural people's access to markets is now one of its top priorities, and the proportion of IFAD‐supported projects that include work on market access has increased dramatically (increasing from 3% to more than 75% over the last two decades).

Correspondingly, evidence of the effects of interventions addressing farmers’ access to output markets has expanded in recent years. Bold et al. (2022) present one example of an experiment examining the effects of an intervention in Uganda coupling agricultural extension services with a market access component (where an agro‐trading company committed to offering to buy farmers’ quality maize at a premium). Other examples have also examined the effects of the construction of roads, increasing the provision of market information, and using technology to create more unified agricultural markets (e.g., see Khandker & Koolwal, 2010; Levi et al., 2020; Svensson & Yanagizawa, 2009).

Some efforts to synthesise evidence on the effects of agricultural market access interventions exist. For example, systematic reviews by Hine et al., (2016, 2019) and Ludwig et al. (2016) find rural road investment is associated with increased income, poverty reduction, employment, agricultural output and sales. Aker et al. (2016) provide a literature review on agricultural information technology initiatives (such as information interventions using mobile phones) and conclude that evidence of their effects on farmers’ behaviour and welfare outcomes is mixed. A systematic review on contract farming by Ton et al. (2017) also suggests that such schemes may increase farmer income but that the effects may be concentrated among wealthier farmers who are better placed to take advantage of these types of arrangements.

However, while the results and implications derived from existing reviews tend to be narrow and deep, they are broadly less accessible to researchers, practitioners, and funders of evidence than might be ideal. They are distributed widely across the literature, often focus on only one particular intervention type and the approaches for evidence selection, appraisal and analysis are rarely comparable. Furthermore, extant reviews are also fast becoming dated (e.g., the systematic search by Ton et al. (2017) was last completed in 2015). Some reviews include a limited sample of studies (e.g., Bellemare and Bloem's [2018] review on contract farming restricts the search for the literature to published academic journal articles). Also, many reviews do not use transparent and systematic methods for synthesis or formal approaches for critical appraisal, which means they cannot be considered systematic reviews as defined by Campbell Collaboration (such as Aker et al., 2016).

We intend to provide a new and updated systematic review, bringing together a summary of the evidence for the five focal agricultural market access intervention types indicated above. In doing so, it will provide a new synthesis in areas where there have been relatively limited efforts to synthesise evidence using systematic approaches (such as output market information and storage infrastructure interventions and initiatives creating new or alternative marketplaces) and update and expand reviews where there are some existing efforts (e.g., concerning infrastructure and contract farming).^4^

## 2 OBJECTIVES

The purpose of this review is to identify, assess and synthesise evidence of the effects of interventions addressing farmers’ access to output markets. To address this objective, we intend to answer the following questions:

(1) What does evidence indicate about the direction and magnitude of the effects of output market access interventions on agricultural, socio‐economic and food and nutrition security outcomes?
(2) How does the distribution of effects differ across different contexts, interventions, and outcomes and do the effects of interventions differ between sub‐groups of the population (e.g., according to sex, race, ethnicity, age, socio‐economic status, type of produce, farm size, etc.)?
(3) What is the risk of bias of studies on the effects of output market access interventions on farmers’ on farmers’ agricultural, socio‐economic and food and nutrition security outcomes in LMICs?
(4) What evidence is available in studies included in the review on program costs and their cost‐effectiveness?
(5) Where do gaps exist in the literature and how can future research enrich the evidence on the effects of interventions designed to improve access to agricultural markets in LMICs?

## 3 METHODS

### 3.1 Criteria for considering studies for this review

Next, we outline the inclusion and exclusion criteria, which define the factors determining whether a particular study will ultimately be included in the review.

#### 3.1.1 Types of studies

We will include studies using experimental and quasi‐experimental study designs to measure a change in outcomes that is attributable to an intervention. This is accomplished by identifying a counterfactual, which provides evidence about what would have happened in the absence of the intervention. The counterfactual may be inferred from a specific group of observations that do not receive the intervention (as in a randomised controlled trial [RCT]), or receive an alternative intervention, or may be constructed by researchers (as in propensity score matching or interrupted time series). This means studies may apply a wide range of potential designs, such as randomised controlled trials, regression discontinuity designs, instrumental variables, fixed effects regressions, interrupted time series models, matching methods, and the synthetic control method (see Supporting Information: Appendix 1 for a comprehensive list of included study designs). These study designs are widely recognised as being capable of establishing causal relationships between interventions and outcomes when carefully and credibly executed (Aloe et al., 2017; Fenton Villar & Waddington, 2019; Gertler et al., 2011; Reeves et al., 2017; Waddington et al., 2023).

We will not exclude studies based on the comparison condition of a control group. A study's control group may consist of observations subject to no intervention, on a wait‐list, or a member of an alternative intervention or condition. However, we will exclude studies that only use simulation or forecast models, ex‐ante impact assessments or scenario analyses, as well as evaluations and case studies that do not satisfy the methodological conditions described above. We will also exclude efficacy studies, feasibility studies, acceptability studies, and literature reviews and systematic reviews will not be included as primary studies.

The nature of many of the interventions we intend to study, particularly large infrastructure interventions and widespread information initiatives, may create spillover effects and market equilibrium outcomes. For example, Hildebrandt et al. (2020) provide evidence of spillovers from a price alert system in Ghana. We will document where studies present both partial and general equilibrium effects of interventions (e.g., Yanagizawa‐Drott & Svensson, 2012) or control for this issue via a study design (e.g., Bergquist & McIntosh, 2021).

#### 3.1.2 Types of participants

We will include studies of the effects of interventions consisting of participants residing in LMICs. We will use the World Bank income status classification for defining LMICs (see Supporting Information: Appendix 2) and studies will be classified according to their status in the year the intervention began.^5^ Some studies can include evidence of the effects of interventions implemented in more than one country. A study including interventions from multiple countries will be included if results are provided for LMIC and HIC countries separately.

#### 3.1.3 Types of interventions

We will include interventions that aim to strengthen farmers’ output market access using one or more of the five types of interventions we describe above. We will consider both farmers with already access to markets (commercialised farmers) and subsistence farmers. This list of included interventions is not an exhaustive list of potentially relevant output market access interventions but provides a selection of key strategies. The selection of interventions included is informed by funder interests and expert advice.

As also noted in our discussion above, there are variations in the way interventions may appear in practice. Table 1 characterises further the different ways these intervention types may be sub‐categorised, and our analysis is designed to consider variation within intervention types, as well as between them, where there is sufficient evidence to do so. However, at this point, we do not intend to be too prescriptive about the sub‐categories of interventions included or those we will group together for the analysis. Practitioners are often highly industrious in finding innovative ways to address problems. We are adopting an iterative approach, where we will update these sub‐categories of interventions again once we have identified the full population of studies included in the review. The sub‐categories of interventions we present in Table 1 provide a starting point for this exercise based on expert advice and knowledge of existing studies from our initial searches to develop our search strategy (discussed below). We will perform a further mapping exercise analysing interventions for any other notable affinities that can be gleaned from the available information about the interventions, as well as the differences between them.

**Table 1:** List of included interventions and sub‐categories of interventions.

Intervention Type	Intervention sub‐categories	Definition
Farm to market transport infrastructure interventions	Domestic transport infrastructure	Initiatives that improve infrastructure primarily used for domestic transport, such as rural roads, bridges, waterways and river transport.
	Export transport infrastructure	Initiatives that improve infrastructure used primarily to export produce to foreign markets, such as at ports, border crossings or international distribution facilities.
Output market information interventions	Provision of output market information (mobile and internet)	Interventions that provide farmers with output market information. The mode of information dissemination must include mobile (e.g., via sms, phone) and internet (e.g., via apps, website, etc.) technologies.
	Provision of output market information (other forms of communication)	Interventions that provide farmers with output market information. The mode of information dissemination can include other more traditional forms of communication and technology (e.g., radio, peer networks, etc.).
	Investments in information and communication technologies infrastructure	Interventions that increase access to technologies required to access market information. This may include investments in mobile phone services, internet or broadband access, and the basic provision of electricity.
Initiatives creating new marketplaces & alternative marketing opportunities	Online commodity exchanges and mobile‐based marketplaces	Interventions improving connections between market participants by creating new or improved marketplaces using information technologies, e.g., via online commodity exchanges, apps or mobile services.
	Alternative physical marketplaces	Interventions improving connections between market participants by creating physical alternative marketplaces, such as physical hubs linking farmers directly with private companies.
	Arranged or curated offers from buyers	Interventions improving connections between market participants by curating commitments or stable arrangements where traders offer to buy farmers’ produce (e.g., Bold et al., 2022; Maertens et al., 2020).
Contract farming initiatives (inc. out‐grower schemes)	Fixed‐price and price guarantee contracts	Fixed‐price contracts are forward‐looking agreements in which the buyer provides farmers a guaranteed price for the delivery of agricultural produce.
	Production‐management contracts	Production‐management contracts whereby the buyer provides extension services or other technical support for the delivery of agricultural produce.
	Input or credit‐supply contracts	Input or credit‐supply contracts where the buyer provides inputs or credit upfront and deducts the cost at harvest.
	Other contract types	Other types of contractual arrangements between farmers and buyers for the future delivery of agricultural produce.
Improved storage infrastructure interventions	Improved on‐farm storage infrastructure	Interventions that provide technical, logistical, or financial support for improved on‐farm farm storage infrastructure, such as the use of sheds with off‐the‐ground storage.
	Storage deposit systems	Interventions that enable farmers to deposit produce in off‐farm stores, such as warehouse receipt systems.

*Note*: We will include studies examining the effects of households’ participation in contract framing schemes, as well as field experiments providing or offering contract farming as part of an intervention.

Interventions may include activities addressing these issues in isolation or in combination with activities. For example, a farming contract may include both a fixed price agreement and financing for inputs or, alternatively, an intervention may include multiple components consisting of both the provision of market information and a market‐making activity using information technology. They may also include another intervention that is not in itself included in this review, such as agriculture extension services with a market access intervention (as in Bold et al., 2022). Our review will endeavour to record the nuances of intervention arrangements in its description and analysis.

We will include interventions targeting farmers of seasonal (such as grains, pulses, or root and tuber crops, etc.) and permanent crops (such as cocoa, coffee, tea, etc.). This includes horticulture products, and we will also include interventions concerning livestock and animal husbandry, which is the branch of agriculture concerned with animals that are raised for meat, milk, skins, hides, and so forth. However, to limit the overall breadth of the scope of the review, we will not include studies on market access interventions from related sectors (i.e., the fishery or forestry sectors). We add this clarification because some organisations, such as the Food and Agriculture Organization of the United Nations (FAO) and USAID, aggregate these economic activities into a single agriculture sector.

With respect to interventions creating alternative marketing opportunities or new markets, we will exclude public marketing (or commodity) boards, which are set up by a government to regulate the buying and selling of a certain commodity within an area (e.g., see Fung et al., 2020). We will also exclude minimum support price policies (MSPs), which are also recognised as a type of output subsidy and are regularly used by governments to safeguard farmers’ income against crop price falls and ensure a sufficient and balanced production of crops. Furthermore, we will exclude studies examining the effects of establishing traditional agricultural commodity exchanges or programmes promoting post‐harvest practices and technologies, such as drying tarps (e.g., see Magnan et al., 2021). We will also exclude studies that concern certification only, such as UTZ Certified, Fair Trade, and Rainforest Alliance, unless a non‐transferable fixed‐term forward sales contract with a specific firm is offered. Sharecropping arrangements where a tenant farmer is provided with inputs in exchange for an agreed share of the harvest are also excluded.

#### 3.1.4 Types of outcome measures

We will include studies that contain data on outcomes related to the conceptual framework above. This includes immediate outcomes (such as transactions costs, aggregation, use/adoption of improved inputs, technologies and practices, farm investment, access to credit and prices, crop losses), agricultural production outcomes (including measure of yields, volume, quality, produce type), intermediate outcomes (farm sales and income), non‐ and off‐farm income and labour outcomes, and welfare outcomes (total household income and food security).

Table 2 provides further details of each of the outcome types included. This may not be an entirely exhaustive list of potential outcomes for these interventions. There are many channels through which these interventions may operate and some outcomes, such as farmers’ market risk, are also difficult to define or measure in the context of an impact evaluation. Informed by expert advice, this review focuses on a selection of key outcomes which provides good coverage of a broad range of relevant outcomes related to the interventions’ theory of change and the primary welfare outcomes of interest. All outcomes here are relevant for the five interventions types considered in this review. However, we expect the prevalence of outcomes featured in the primary research evaluations to dictate the final outcomes included for each intervention within our framework. We will reflect on the prevalence of these particular outcomes in the systematic review for each intervention type.

**Table 2:** List of included outcomes.

Outcome Type	Outcome	Description
Immediate outcomes	Transaction costs	This includes measures of costs associated with trading produce and that are accrued by farmers (such as transport costs).
	Aggregation	This includes outcomes measuring the incidence of farmer participation or membership of cooperatives and farmer groups.
	Use/adoption of improved inputs, technologies and practices	This includes outcomes measuring the use or adoption of improved inputs, technologies, and practices (such as pesticides, specialised seeds, etc). This also include measures of whether farmers have produce in storage, or the amount of produce in storage.
	Farm investment	This includes measures of the monetary value of farmer investment (spending) on improved farm infrastructure, technologies, inputs, etc.
	Access to credit	This includes outcomes measuring farmers access or use of credit (such as the total amount borrowed).
	Prices	This includes farm‐gate prices, that is, those effectively received by farmers for their produce. Prices may be measured through farmers’ reports of prices, market surveys, and from records of market transactions.
	Crop losses	This includes measures of post‐harvest losses.
Agricultural Production outcomes	Yields	Yields are a measure of agricultural productivity.
	Volume	This includes measures of the quantity or volume of agricultural production or output and share or amount of land cultivated
	Quality	This includes measures of the quality of produce (e.g., Aflatoxin and moisture in stored crops).
	Produce type	This includes outcomes where studies are specifically intending to measure the adoption or increased volume of an alternative variety of crop or produce (e.g., production of specialised crop varieties, production of cash crops, etc.).
Intermediate outcomes	Sales	This includes measures of the proportion, number and the volume of produce sold by farmers.
	Farm income	This includes measures of income generated from farm activities.
Off‐farm outcomes (Agriculture only)	Off‐farm income	This includes measures of income generated from off‐farm agriculture‐related activities that occur beyond the farm owned by the household (e.g., off‐farm wage work in agriculture).
	Labour market outcomes	This includes measures of employment outcomes and number of hours worked for off‐farm agriculture‐related activities that occur beyond the farm owned by the household (e.g., off‐farm wage work in agriculture).
Non/off‐farm outcomes	Non/off‐farm income	This includes measures of income generated from non/off‐farm activities.
	Labour market outcomes	This includes measures of employment outcomes and number of hours worked (not on a farm owned by the household).
Welfare outcomes	Total income and wealth	This includes measures of total household income and other measures of socio‐economic status (such as total household expenditure and asset or wealth indices).
	Food and nutrition security	Food and nutrition security concerns the state of having reliable access to a sufficient quantity of affordable, nutritious food. We will include indices of food and nutrition security, composite scores of the extent to which households have food to meet basic dietary needs, measures of nutritional intake and food consumption and outcomes based on whether households report they have sufficient food.

Interventions investing in societal infrastructure (such as roads and bridges concerning farm to market interventions or internet and electricity infrastructure under information interventions) may also have several possible channels explaining their effects on welfare outcomes. For example, other possible channels beyond the hypothesised effect on the agricultural sector may include improved access to health care, education, other labour markets, and so forth. Reflecting the theme of the review, since in this instance we are interested in understanding their effects through the agricultural development channel, we will include welfare, non‐ and off‐farm income and labour market outcomes, but only from studies that also report agricultural production outcomes, intermediate outcomes, or off‐farm (agriculture only) outcomes. In our analysis, we intend to examine the statistical correspondence between the estimated effects on agricultural outcomes and these other outcomes (e.g., confirming that evidence indicates infrastructure interventions improve both agricultural outcomes and welfare outcomes).

#### 3.1.5 Other inclusion and exclusion criteria

**Language:** Studies published in any language will be included, although the search terms used will be in English only.

**Publication date:** We will include studies published in 2000 or later. This restriction is necessary to keep the review's search feasible within project resources. Furthermore, analyses indicate that although there were IEs published in international development before 2000, the number is relatively small, especially in the agriculture sector (see Sabet & Brown, 2018).

### 3.2 Search methods for identification of studies

To identify relevant literature, we will conduct a comprehensive search for eligible published and unpublished studies. Our search strategy has been developed in collaboration with an information specialist and with reference to guidance in Kugley et al. (2017). We have developed a set of English search terms which we will use in a wide array of electronic academic and institutional databases. We will also conduct citation tracking, publish a blog presenting a public call for papers, and we will contact key experts and organisations to identify additional studies.

#### 3.2.1 Electronic searches

To identify relevant studies for our review, we have developed a set of English search terms and a search strategy in collaboration with an information specialist. Our search terms combine Boolean terms with a list of keywords related to the review's inclusion criteria (see Supporting Information: Appendix 3). We will use and adapt these terms to search electronic databases and institutional websites with sufficient search functionality.

As suggested by Sandieson (2006), we have utilised pearl‐harvesting techniques to develop our list of key search terms. This involves reviewing and identifying keywords from our inclusion criteria and known studies relevant to the review (see Supporting Information: Appendix 4 for further details). We identified an initial sample of relevant studies by consulting with members of the project's expert advisory group and searching the results of a selection of existing reviews (see Supporting Information: Appendix 5). We also performed targeted searches of 3ie's Development Evidence Portal (DEP), which is currently the world's most expansive repository of rigorous evidence on what works in international development. We used the DEP's intervention search filters, which index studies using a standardised vocabulary for classifying interventions (see Kozakiewicz et al., 2021), to help to identify a sample of studies listed in the portal that are most relevant to our review. Supporting Information: Appendix 6 provides a list of the initial list of studies identified for peal‐harvesting and developing our search strategy.

We have also compiled a list of databases and websites we will search for relevant evaluations and studies (see Table 3). To reduce the risk of publication bias, these sources have been selected to cover a range of publication types, including journal articles, working and discussion papers, conference proceedings, thesis and dissertations, and institutional reports. We have identified relevant sources by consulting an information specialist, the project's expert advisory group, and systematic reviews on included or related interventions recording databases including impact evaluation evidence (including Hine et al., 2016, 2019; Ludwig et al., 2016; Oya et al., 2017, 2018; Ton et al., 2017; Waddington et al., 2014).

**Table 3:** Table of electronic databases and websites to be searched.

Type	Sources
Databases and search engines	Scopus CAB Abstracts Web of Science^a^ Agricola Econlit EBSCO Discovery Service^b^ International Bibliography of the Social Sciences Sociological Abstracts ProQuest dissertations and theses database
Institutional websites and repositories	3ie Development Evidence Portal Action Against Hunger ActionAid Abdul Latif Jameel Poverty Action Lab (J‐PAL) African Development Bank (AfDB) Agricultural Technology Adoption Initiative (ATAI) Asian Development Bank (ADB) Centre for Agricultural Bioscience International (CABI) Danish International Development Agency (DANIDA) Australian Government Department of Foreign Affairs and Trade (DFAT) Development Experience Clearinghouse (USAID) Food and Agriculture Organization (FAO) Farm Africa Feed the Future Innovation Lab for Markets, Risk & Resilience Deutsche Gesellschaft für Internationale Zusammenarbeit (GIZ) International Fund for Agricultural Development (IFAD) International Food Policy Research Institute (IFPRI) Innovations for Poverty Action (IPA) Inter‐American Development Bank (IADB) Netherlands Ministry of Foreign Affairs Norwegian Agency for Development Cooperation (NORAD) Oxfam Policy and Practice Research for Development: FCDO The Expert Group for Aid Studies (EBA) World Food Programme (WFP)

*Note*: Details of the search pathway and any filters applied to websites will be recorded and published in Supporting Information with the final report.

^a^ Including Web of Science Core Collection (Social Sciences Citation Index (SSCI),Science Citation Index Expanded (SCI‐EXPANDED), Conference Proceedings Citation Index—Science (CPCI‐S), Conference Proceedings Citation Index—Social Science & Humanities (CPCI‐SSH), Emerging Sources Citation Index (ESCI).

^b^ Including GreenFILE, Academic Search Complete, Science Direct, AGRIS, RePEc, World Bank e‐Library.

While some websites and databases have reasonably well‐developed search functions, some do not support complex search strings or allow for the direct export of materials, and others must be browsed by keywords or even browsed in their entirety. We will customise our general search strategy according to the functionality of each database and website we search (using the website's thesaurus or keyword index if necessary to identify the most appropriate vocabulary). We will consult an information specialist who will help to troubleshoot problematic sources, as well as advise on the best ways of conducting targeted searches. We will document the literature search process and any necessary changes to the search strategy for each source.

#### 3.2.2 Citation tracking

For the studies included in the review, we will also perform backward and forward citation tracking (Greenhalgh & Peacock, 2005). Backward citation tracking consists of screening the reference lists or bibliography of included studies for other eligible studies cited in the text. Here we will perform hand searches and manually record the number of studies screened. Forward citation tracking involves searching for eligible studies that cite the originally included study. For forward citation tracking, we will utilise Google Scholar.

#### 3.2.3 Searching other resources

We will supplement these searches by contacting key researchers and organisations working on issues related to this review and we will engage our expert advisory group for suggestions concerning other relevant studies. Finally, we will search the included studies of other related evidence maps and reviews. Supporting Information: Appendix 5 provides a provisional list of relevant maps and reviews.

### 3.3 Data collection and analysis

#### 3.3.1 Selection of studies

After collating and de‐duplicating records from our literature search, we will perform a two‐stage selection process where trained reviewers will assess studies against the review's inclusion and exclusion criteria.

##### De‐duplication of records in the literature search

First, the search results from databases and search engines will go through an initial round of de‐duplication in R using the *synthesisr* package. Results will then be imported to the EPPI‐Reviewer software (Thomas et al., 2022), where EPPI's de‐duplication functionality will also be applied as a second check for duplicate records. For studies identified through forward citation tracking, we will also de‐duplicate records where it is possible to export the bibliographic information of a records reference list and upload this to EPPI. Any additional relevant studies identified through suggestions from our expert advisory group, study authors and backward citation tracking will be manually captured in EPPI Reviewer where they cannot be identified in our search results.

###### Stage 1—Title and abstract screening

After de‐duplicating records from our literature search, the first stage of the selection process will consist of screening the information available in study titles and abstracts against the inclusion and exclusion criteria. Trained reviewers will independently screen studies and those that are relevant and meet the inclusion criteria will be flagged for a full‐text review in the second stage of the selection process. We will exclude all studies that do not meet the inclusion criteria. However, if a study's title and abstract do not provide sufficient information to determine its relevance to the criteria, we will review its full text (see Stage 2 below). The reviewers will exclude studies based on a prioritisation and sequential exclusion approach (Saif‐Ur‐Rahman et al., 2022). We will present the exclusion criteria as a series of questions to the reviewers and arrange them in the sequential order further described in Table 4.

**Table 4:** Table of exclusion criteria.

Priority order	Question	Excluded if the answer is
1.	Is the study a lab or efficacy study?	Yes
2.	Does the study evaluate an intervention, policy, programme, project?	No
3.	Are participants living in a high‐income country at the time the intervention began (see Section 3.1‐2)?	Yes
4.	Does the study include a study design that is consistent with the review's inclusion criteria (see Section 3.1‐1)?	No
5.	Has the study been published before the year 2000?	Yes
6.	Does the study include an intervention that is consistent with the review's inclusion criteria (see Section 3.1‐3)?	No
7.	Does the study include an outcome that is consistent with the review's inclusion criteria (see Section 3.1‐4)?	No

*Note*: If insufficient information is available to confidently answer a question, reviewers will proceed to the next question without excluding the study. The excluded lab studies do not exclude studies using lab‐in‐the‐field experiments to measure changes in behavioural outcomes in the context of an evaluation of an intervention (e.g., see Armand et al., 2019).

Reviewers will be trained to use EPPI‐reviewer for screening purposes, and they will also undergo theory‐ and practice‐based training on how to consistently apply the reviews inclusion/exclusion criteria to studies. They will perform this training in groups and use a ‘training set’ of studies. Following their training, reviewers will independently double screen the title and abstracts of the same records until we find they reach a 85% inter‐rater reliability (consistency) rate for include/exclude decisions. We will allocate pairs of reviews sequential batches of the same 200 records for screening. At the end of each batch, we will calculate the inter‐rater reliability rate. With the aim of establishing a high level of inter‐rater reliability in the following rounds, we will arrange a meeting between reviewers and a member of the core team to discuss disagreements in their application of study eligibility criteria.

Once reviewers have reached the required inter‐rater reliability rate, they will advance to the main tranche of title‐abstract screening where they will screen records entirely independently. During the independent screening phase, we will follow a ‘safety first’ approach whereby, if a reviewer is uncertain about whether a study should be included or excluded, they can request a second opinion from another reviewer. Periodic meetings will be held by members of the core team to address studies flagged for a second opinion and make any refinements to the screening approach and provide further guidance to reviewers if required.

The search for literature is likely to identify many thousands of studies, some being more relevant to our review than others. We aim to utilise the machine learning capabilities in EPPI‐Reviewer 4 to expedite the title and abstract screening process using its ‘classifier’ functionality (see O'mara‐Eves et al., 2015; Thomas et al., 2011). An initial version of this classifier model has been developed using data on past inclusion and exclusion decisions from 3ie's DEP. This model will be applied to the records identified from databases with exportable search results and each record will be given a (pseudo) propensity score indicating the estimated likelihood of it being an LMIC‐focused impact evaluation. We will screen all records where the upper‐bound estimates from the classifier model are greater than 10 per cent and screen a sample of records with scores between 0 and 10 per cent to check the efficacy of the model.^6^

We will also utilise past inclusion and exclusion decisions from 3ie's ongoing evidence surveillance project. Having searched and screened studies for over a decade for its Development Evidence Portal (DEP) and other evidence synthesis projects (including systematic reviews and evidence gap maps), it has recorded a repository of studies that have already been screened and are excludable (e.g., because the study does not contain an includable study design or country relevance). We will compile and use this repository of previously screened studies to exclude studies identified by our search.

###### Stage 2—Full text screening and study selection

In the second stage of the study selection process, using two independent reviewers we will double screen all studies flagged for a review using each manuscript's full text. We will resolve any disagreements between the reviewers concerning a study's inclusion through a discussion with a third core review team member, and the input of an additional core reviewer if necessary. Again, we will follow the sequential exclusion criteria outlined above in determining an inclusion or exclusion decision for each study and reviewers will also undergo theory‐ and practice‐based training on how to consistently apply the reviews inclusion/exclusion criteria to studies.

We also expect to identify multiple papers related to the same study. We will use the ‘linked studies’ functionality of EPPI reviewer to note the main study and other linked studies. The main study will be used for data extraction and the linked studies will be stored to help any required search for further or missing information. Linked studies will also be used when they report on outcomes relevant to this review that are not reported in the main study. To identify the main study, priority will be given to journal articles and, in the case of multiple reports, working papers or articles, the most recent one will be selected.

#### 3.3.2 Data extraction and management

We will extract the following descriptive, methodological, quantitative, and cost and other qualitative data from each study included in the review using standardised data extraction forms (provisional forms are provided in Supporting Information: Appendix 7):

- Descriptive data including authors, publication date and status, as well as other information to characterise the study including country, type of intervention and outcome, and intervention design.
- Methodological information on study design, analysis method, and type of comparison (if relevant).
- Quantitative data for outcome measures, including outcome descriptive information, sample size in each of the intervention and comparison groups, outcomes means and SDs and test statistics (e.g., *t* test, *F* test, *p* values, 95% confidence intervals).
- Cost data.

Descriptive data, methodological information and cost data will be single coded by a trained reviewer and checked for agreement by another one. Two trained reviewers will independently code the quantitative data and any disagreement will be resolved through discussion with a third reviewer (who must be a core team member).

#### 3.3.3 Assessment of risk of bias in included studies

We will assess the risk of bias in included studies using 3ie's risk of bias tool (see Supporting Information: Appendix 10). This examines both the internal validity and statistical conclusion validity of experimental and quasi‐experimental impact evaluation designs (see Waddington et al., 2012). Two reviewers will undertake the risk of bias assessment independently. If there are disagreements, we will resolve them by discussion and the involvement of a third reviewer (who must be a member of the core team). We will compile a risk of bias assessment for each estimate we extract. This reflects that reflecting estimates on different outcomes in the same study may score differently in the assessment.

We will assess the risk of bias based on the following criteria, coding each estimate as ‘Yes’, ‘Probably Yes’, ‘Probably No’, ‘No’ and ‘No Information’ for each domain:

- Factors relating to baseline confounding and biases arising from differential selection into and out of the study (e.g., assignment mechanism).
- Factors relating to bias due to missing outcome data (e.g., assessment of attrition).
- Factors relating to biases due to deviations from intended interventions (e.g., performance bias and survey effects) and motivation bias (Hawthorne effects).
- Factors relating to biases in outcomes measurement (e.g., social desirability or courtesy bias, recall bias).
- Factors relating to biases in reporting of analysis.

We will report the results of the assessment for each of the assessed criteria for each estimate. In addition, we will use the results of the risk of bias assessments to produce an overall rating for each study as either ‘High risk of bias’, ‘Some concerns’ or ‘Low risk of bias’, drawing on the decision rules in RoB2.0 (Sterne et al., 2019), rating studies as follows:

- ‘High risk of bias’: if any of the bias domains were assessed as ‘No’ or ‘Probably No’.
- ‘Some concerns’: if one or several domains were assessed as ‘No Information’ and none were ‘No’ or ‘Probably No’.
- ‘Low risk of bias’: if all of the bias domains were assessed as ‘Yes’ or ‘Probably Yes’.

We will provide a description in our analysis of the outcomes of our assessment of reliability of included studies, and we also intend to explore whether there are systematic differences in estimated effects between primary studies with different risk of bias. We will conduct sensitivity analysis to assess the robustness of the results to the risk of bias associated with included studies (discussed below).

#### 3.3.4 Measures of treatment effect

In this review, we intend to examine the effects of output market access interventions. An effect size (or treatment effect) expresses the direction and magnitude of the difference in outcomes between groups of observations, such as the difference in outcomes between observations in the intervention and comparison groups (Borenstein et al., 2009a; Valentine et al., 2015).

However, effect sizes presented in empirical studies are rarely independent of the scale or unit of the outcome in the study and the scale or unit of the outcome is rarely directly comparable across studies. For these reasons, to facilitate cross‐study comparisons of the magnitudes of studies effects in our analysis, we will extract data from each study to calculate standardised effects sizes. We will choose the appropriate formulae for standardised effect size calculations in reference to, and dependent upon, the data provided in the included studies and the outcome type (see Supporting Information: Appendix 8 for details of the effect size formulae sheet).

If different outcome types exist under the same outcome construct (e.g., binary measure of employment and a measure of the number of hours worked off‐farm), for comparability of estimated effect sizes, we will we convert estimates to the most common standardised metric. We will use common transformations outlined in Borenstein et al. (2009a) for converting between different measures of standardised effects.

#### 3.3.5 Criteria for determination of independent findings

It is important our analysis accurately captures and reflects on co‐dependencies between study estimates. This is because standard meta‐analytic methods assume effect size estimates are independent and failure to qualitatively recognise estimates are derived from the same intervention or study can distort (inflate) our perceptions of the availability of evidence.

Dependent effect sizes can arise in several circumstances. For example, co‐dependencies between estimates can arise when several publications stem from one study, or several studies are based on the same data set. Some studies might have multiple treatment arms that are all compared to a single control group. Other studies may report outcome measurements from several time points or use multiple outcome measures to assess related outcome constructs. All such cases yield a set of statistically dependent effect size estimates (Borenstein et al., 2009b).

We will assess the extent to which relationships exist across the studies included in the review. We will avoid double counting of identical evidence by linking papers before data analysis. We will utilise information provided in the studies included to help support these assessments, such as sample sizes, programme characteristics and key implementing and/or funding partners. Where we have several publications reporting on the exact same effect, one main study will be used for data extraction and the linked studies will be stored to help any required search for further or missing information. To identify the main study, priority will be given to journal articles and, in the case of multiple reports/working papers, the most recent one will be selected.

We will extract effects reported across different interventions, outcomes and subgroups within a study. We will address dependent effect sizes using data processing and selection techniques. We will utilise several criteria to select one effect estimate per outcome per study (further details of the criteria determining effect estimate selection are available in Supporting Information: Appendix 9).

#### 3.3.6 Unit of analysis issues

Unit of analysis errors can arise when the unit of allocation of a treatment is different to the unit of analysis of effect size estimate, and this is not accounted for in the analysis (e.g., by clustering *SE*s at the level of allocation). We will assess included studies for the prevalence of these issues and, where they exist, account for them by adjusting the reported *SE*s according to the following formula (Hedges, 2009; Higgins et al., 2020):

$${\left(d\right)}^{\prime }=\left(d\right)\;\cdot \;1+\left(m-1\right)c,$$

where *d* is the effect size, *m* is the average number of observations per cluster and *c* is the intra‐cluster correlation coefficient. If the included studies use robust Huber‐White *SE*s to correct for clustering, we will calculate the *SE* of *d* by dividing *d* by the *t*‐statistic on the coefficient of interest. We will search for an appropriate ICC in the literature and if this is not available we will we assume the ICC to be 0.05, as also described in Waddington et al. (2014).

#### 3.3.7 Dealing with missing data

In instances where there is missing or incomplete data, we will make every effort to contact study authors to obtain the required information. If we are unable to obtain the necessary data, we will report the characteristics of the study but state that it could not be included in the meta‐analysis or reporting of effect sizes due to missing data. In line with recommendations on collating data in systematic reviews from study authors (see Mullan et al., 2009), we will report the number of studies for which authors were contacted, the information requested, any important details of the method of eliciting information, and the response of authors to the request. When pertinent, we will also report the impact that information obtained from authors has on the results (i.e., using sensitivity analyses discussed below).

#### 3.3.8 Data synthesis

To synthesise the effects of market access interventions, we will combine a narrative synthesis of study findings with a meta‐analyses of interventions effects.

We will include studies in the same meta‐analysis when we identify two or more effect sizes using a similar outcome construct, the same intervention type, and where the type of comparison group is judged to be similar across the studies. This is similar to the approach taken by Wilson et al. (2011). Where there are too few studies, or the included studies are considered too heterogeneous in terms of interventions or outcomes, we will present a narrative discussion of individual effect sizes alone.

Because heterogeneity exists in theory due to the variety of interventions and contexts that could be included in the review, we will use inverse‐variance weighted, random effects meta‐analytic models (Higgins et al., 2020). We will use the metafor package (Viechtbauer, 2010) in R software to conduct the meta‐analyses (R Core Team, 2020).

The narrative synthesis of study findings will provide information on the relevant information on the associated factors facilitating and moderating the effects. This information will be synthesised to possibly draw conclusions beyond the study level and design learnings on the necessary conditions for positive effects to occur. This analysis will closely follow the meta‐analysis results and interpretations and the subgroup and heterogeneity analysis described in Section 3.3‐10.

We will also examine the statistical correspondence between welfare outcomes and other more proximate outcomes in the conceptual model. For example, examining the correspondence in effects between immediate and agricultural outcomes and welfare outcomes. We will do this through descriptive analysis and, where possible, regression analysis and fuzzy Qualitative Comparative Analysis (QCA) (Thomas et al., 2014; Ton et al., 2017). We will also document the results and correspondence of estimated effects for studies that provide insights through general equilibrium analysis, as well as partial effects.

#### 3.3.9 Assessment of reporting biases

If meta‐analysis is feasible, we will assess reporting biases in the literature using a rank correlation test (see Begg & Mazumdar, 1994). We will also use a regression test (Sterne & Egger, 2005), using the standard error of the observed outcomes as predictor, to check for funnel plot asymmetry.

#### 3.3.10 Subgroup analysis and investigation of heterogeneity

In our analysis, we intend to examine and discuss the distribution of estimated effects across intervention and outcome types. We will also statistically assess heterogeneity by calculating the *Q* statistic, *I* ^2^, and *τ* ^2^ to provide an estimate of the amount of variability in the distribution of study effects sizes (Borenstein et al., 2009a). We will complement this assessment with a graphical analysis using forest plots and, whenever feasible, we will conduct moderator analyses using meta‐regression analysis to investigate sources of heterogeneity.

Following the PROGRESS‐PLUS approach (Oliver et al., 2017), we will assess moderators falling into three broad categories of extrinsic, methodological and substantive characteristics. Examples of these categories include:

- Extrinsic characteristics: funder of the study (e.g., NGO vs. private sector vs. government investments), publication type, publication date.
- Methodological characteristics: study design, risk of bias, length of follow‐up, types of outcome measures.
- Substantive characteristics: participant characteristics (gender, age, socio‐economic status, education, land ownership, farm size), context (geographical setting, crop type), intervention type, intervention features, type of implementing agency.

We will use random effects meta‐regression to investigate the association between moderator variables and heterogeneity of treatment effects (Borenstein et al., 2009a), and subgroup analyses to investigate heterogeneity by treatment subgroups (e.g., men and women, poor and non‐poor, and so on). If these strategies are not possible (e.g., if we do not have sufficient number of studies or data), we will discuss and explore the factors which may be driving the heterogeneity of results narratively by conducting cross‐case comparisons (Miles & Huberman, 1994).

#### 3.3.11 Analysis of intervention costs

We will collate and analyse any cost data reported in the set of studies included in our review or from any documents they reference. Following Shemilt and Mugford (2009), relevant studies will include full economic evaluations (e.g., cost–benefit, cost‐effectiveness or cost‐utility analyses), partial economic evaluations (e.g., cost analyses, cost‐comparison studies, cost‐outcome descriptions), or any other documentation reporting the costs associated with included interventions. Cost information will be tabulated and synthesised narratively with descriptions of details of the different approaches used to derive intervention costs also provided. Estimates show only around 15% of impact evaluations report information about intervention costs (see Brown & Tanner, 2019). Reflecting that it is likely that information on intervention costs will be limited, where possible, we will contact study authors and funders of interventions to try to retrieve further information. We will also collate any additional cost‐effectiveness or other types of economic evaluations identified from our literature search.

#### 3.3.12 Sensitivity analysis

We will conduct sensitivity analysis to assess whether the results of the meta‐analysis are sensitive to the removal of any single study. We will do this by removing studies from the meta‐analysis one‐by one and assessing changes in results. We will also assess the sensitivity of our results to the inclusion of studies with a high risk of bias studies by removing these studies from the meta‐analysis and comparing results to the main meta‐analysis results. We will examine the sensitivity of results to the inclusion of specific outcome measures (e.g., limiting meta‐analysis to the preferred measure of an outcome). We will also assess the sensitivity of our result to the inclusion of data obtained directly from study authors (as discussed above).

Furthermore, we will assess the sensitivity of our results to outliers. We will use studentised residuals to examine whether studies estimated effects may be outliers (Viechtbauer & Cheung, 2010) and studies with a studentised residual larger than the 100×(1−0.05/(2×k))th percentile of a standard normal distribution will be considered potential outliers.

It is possible that we may also use both bivariate and multivariate (or partial) effects for calculating standardised effect sizes. A partial effect size is based on a regression coefficient measuring the treatment effect ‘holding all other variables constant’ and is, therefore, measuring a different quantity to a bivariate relationship. Our standardised effect sizes are only strictly comparable in studies using a common model (Keef & Roberts, 2004). However, only using bivariate effect sizes to calculate standardised effects would not be suitable in this context due to the likely high risk of bias this may cause quasi‐experimental study designs that control for selection bias (Waddington et al., 2014). We will use sensitivity analysis to examine systematic differences in partial and bivariate effects (omitting them from the analysis or controlling for these characteristics in a meta‐regression).

Finally, not all multivariate models control for the same covariates and nor should models estimated for different study designs using data collected in different contexts necessarily do so. The risk of bias assessment evaluates likely specification errors and the sensitivity analysis omitting high risk studies (discussed above) should capture most of these issues. Otherwise, we assume the possible resulting multicollinearity issues are inconsequential (see Waddington et al., 2014).

#### 3.3.13 Analysis of intervention costs

We will collate and analyse any cost data reported in the set of studies included in our review or from any documents they reference. Following Shemilt and Mugford (2009), relevant studies will include full economic evaluations (e.g., cost–benefit, cost‐effectiveness or cost‐utility analyses), partial economic evaluations (e.g., cost analyses, cost‐comparison studies, cost‐outcome descriptions), or any other documentation reporting the costs associated with included interventions. Cost information will be tabulated and synthesised narratively with descriptions of details of the different approaches used to derive intervention costs also provided. Estimates show only around 15% of impact evaluations report information about intervention costs (see Brown & Tanner, 2019). Reflecting that it is likely that information on intervention costs will be limited, where possible, we will contact study authors and funders of interventions to try to retrieve further information. We will also collate any additional cost‐effectiveness or other types of economic evaluations identified from our literature search.

## CONTRIBUTIONS OF AUTHORS

Content: Paul Fenton Villar, Tomasz Kozakiewicz and Shannon Shisler. Systematic review methods: Paul Fenton Villar and Shannon Shisler. Statistical analysis: Shannon Shisler and Paul Fenton Villar. Information retrieval: Sarah Young, Paul Fenton Villar, Tomasz Kozakiewicz, Vinitha Bachina.

## DECLARATIONS OF INTEREST

There are no reported conflicts of interest.

## PRELIMINARY TIMEFRAME

Our tentative date for submission of the systematic review is December 2023.

## PLANS FOR UPDATING THIS REVIEW

The core team will explore opportunities for funding for updates and extensions of this review upon submission of the final report.

## Supporting information

Supporting information.

## Footnotes

1 A multistakeholder umbrella program managed by Food and Agriculture Organization of the United Nations (FAO).

2 However, there is also significant debate about whether traders are exploitative or whether we simply are not pricing in their services or costs correctly (see Dillon & Dambro, 2017).

3 Outgrower schemes refer to arrangements involving public enterprises, parastatals, government agencies or NGOs rather than private entities (Bellemare & Bloem, 2018).

4 We initially considered combining a synthesis of intervention types without systematic review evidence with a review of reviews for interventions with existing systematic review evidence. However, we determined that existing systematic reviews warranted updating to present the most up‐to‐date evidence and, in the process of updating the reviews, standardising the approaches of study selection, analysis, critical appraisal and effect size calculations would increase the comparability of results across different intervention types.

5 We will also include studies from countries that have had a high‐income status for only 1 year before reverting to LMIC status. At the time of writing this protocol, this exception only applies to Argentina (2014, 2017), Venezuela (2014), Mauritius (2019) and Romania (2019).

6 This screening approach is similar to the procedures described by Sabet and Brown (2018) during their empirical testing of this type of machine learning technology using literature from the social sciences. In this instance, propensity score estimates derive from 10 iterations (or re‐builds) of the classifier model using the DEPs data. This provides upper‐ and lower‐bound estimates of the estimated propensity score for each record.
