# Supplementary material for: PROTOCOL: The effects of agricultural output market access interventions on agricultural, socio‐economic and food and nutrition security outcomes in low‐ and middle‐income countries: A systematic review
Source: Campbell Syst Rev. 2023 Aug 21;19(3):e1348. doi: 10.1002/cl2.1348 (PMC10442606; doi:10.1002/cl2.1348)
Supplement: Supplementary file 1 — Supporting information. [file CL2-19-e1348-s001.docx]

**Evidence on the effects of agricultural output market access interventions on agricultural, socio-economic and food and nutrition security outcomes: A systematic review**

Online Appendix

​**Table of Contents**

[Appendix 1. Further information on included study designs 2](#_Toc1507100939)

[Appendix 2. List of included low- and middle-income countries 4](#_Toc2095480506)

[Appendix 3. Details of search strategy 7](#_Toc458751455)

[Appendix 4. Details of process of search strategy development 12](#_Toc965814394)

[Appendix 5. List of related evidence maps and reviews 15](#_Toc50002939)

[Appendix 6. The initial sample of studies used for search strategy development 18](#_Toc330299481)

[Appendix 7. Provisional data extraction forms 29](#_Toc477594436)

[The Study information form 30](#_Toc795908096)

[Quantitative data extraction form 33](#_Toc731754637)

[Cost and other qualitative data extraction form 37](#_Toc1192832255)

[Appendix 8. Calculating standardised effects 37](#_Toc350232079)

[Appendix 9. Criteria determining selection of effect estimates for data extraction 41](#_Toc1381282018)

[Appendix 10. Risk of Bias Assessment Tool 45](#_Toc2076087883)

[Tool for Randomised Experiments 46](#_Toc997230283)

[Tool for Quasi-Experimental Designs 52](#_Toc1316154664)

[Additional References 52](#_Toc1781558782)

# **Appendix 1. Further information on included study designs**

We will include studies using experimental and quasi-experimental study designs to measure a change in outcomes that is attributable to an intervention. This includes studies that apply one of the following approaches:

1. Randomised controlled trials (RCTs), with assignment at individual, household, community, or other cluster level, and quasi-RCTs using prospective methods of assignment (such as alternation)
2. Natural experiments with clearly defined intervention and comparison groups, which exploit natural randomness in implementation assignment by decision makers (e.g., public lottery) or random errors in implementation
3. Regression discontinuity designs (RDD) or fuzzy-RDD
4. Instrumental variables (IV)
5. Endogenous treatment-effects models, endogenous switching regression, and other methods synonymous to the Heckman two step model.
6. Difference-in-differences (DID), two-way fixed-effects (TWFE), and two-way Mundlak regressions (TWM).
7. DiD models will include an interaction term between a time and intervention variable in a regression model. They may also regress an intervention variable on an outcome variable measuring the changes in outcomes over time or present a *t*-test comparing changes in outcomes over time between the intervention and control group.
8. TWFE regressions must include time fixed-effects and unit fixed-effects at the level of the intervention (or lower). For example, if the intervention varies at a village level, it must include either village fixed-effects or fixed-effects of a smaller unit, such as households.
9. TWM models should be synonymous with the approach described by Wooldridge (2021). This includes correlated random-effects and pooled OLS regression models that control for unit-specific time averages and time-period specific cross-sectional averages.

g.  Interrupted time series (ITS) models, with or without a contemporaneous comparison group. This includes segmented regressions, where the time-period is divided into pre- and post-intervention segments, and separate intercepts and/or slopes are estimated for each segment.

1. Weighting and matching approaches that control for observable confounding, including non-parametric approaches (e.g., statistical matching, covariate matching, coarsened-exact matching, propensity score matching) and parametric approaches (e.g., propensity-weighted multiple regression analysis).
2. The synthetic control method

# **Appendix 2. List of included low- and middle-income countries**

| **Low- and middle-income countries (L&MICs)** | | | |
| --- | --- | --- | --- |
| Afghanistan | Dominican Rep. | Liberia | Serbia |
| Albania | Ecuador | Libya | Sierra Leone |
| Algeria | Egypt, AR | Macedonia, FYR | Solomon Islands |
| Angola | El Salvador | Madagascar | Somalia |
| Armenia | Eritrea | Malawi | South Africa |
| Azerbaijan | Ethiopia | Malaysia | South Sudan |
| Bangladesh | Fiji | Maldives | Sri Lanka |
| Belarus | Gabon | Mali | St. Lucia |
| Belize | Gambia, The | Marshall Islands | St. Vin. & Gren. |
| Benin | Georgia | Mauritania | Sudan |
| Bhutan | Ghana | Mexico | Suriname |
| Bolivia | Grenada | Micronesia, FS | Swaziland |
| Bosnia & Herzegovina | Guatemala | Moldova | Syrian Arab Rep. |
| Botswana | Guinea | Mongolia | Tajikistan |
| Brazil | Guinea-Bissau | Montenegro | Tanzania |
| Bulgaria | Guyana | Morocco | Thailand |
| Burkina Faso | Haiti | Mozambique | Timor-Leste |
| Burundi | Honduras | Myanmar | Togo |
| Cambodia | India | Namibia | Tonga |
| Cameroon | Indonesia | Nauru | Tunisia |
| Cape (Cabo) Verde | Iran, I.S. | Nepal | Turkey |
| Central African Rep. | Iraq | Nicaragua | Turkmenistan |
| Chad | Jamaica | Niger | Tuvalu |
| China | Jordan | Nigeria | Uganda |
| Colombia | Kazakhstan | Pakistan | Ukraine |
| Comoros | Kenya | Pap. New Guinea | Uzbekistan |
| Congo, DR | Kiribati | Paraguay | Vanuatu |
| Congo, Republic | Korea, Dem. Republic | Peru | Vietnam |
| Costa Rica | Kosovo | Philippines | W. Bank & Gaza |
| Côte d'Ivoire | Kyrgyz, Republic | Rwanda | Yemen, Republic |
| Cuba | Lao PDR | Samoa | Zambia |
| Djibouti | Lebanon | São Tomé & Prin. | Zimbabwe |
| Dominica | Lesotho | Senegal |  |

| **Former low- and middle-income countries** | | | |
| --- | --- | --- | --- |
| Czechoslovakia | Mayotte (High income: 1990) | Serbia and Mont | Yugoslavia |
| Gibraltar (High income: 2009-2010) | Netherlands Antilles (High income: 1994-2009) | USSR |  |

| **Transitional countries** | | | |
| --- | --- | --- | --- |
| **Name** | | **L&MIC period** | **High-income country period** |
| American Samoa | 1990-present | | 1987-1989 |
| Antigua and Barbuda | 1987-2001; 2003-2004; 2009-2011 | | 2002; 2005-08; 2012-present |
| Argentina | 1987-2013; 2015-16; 2018-present | | 2014; 2017 |
| Aruba | 1991-1993 | | 1987-1990; 1994-present |
| Bahrain | 1990-2000 | | 1987-1989; 2001-present |
| Barbados | 1987-1988;1990-99; 2001;2003-05 | | 1989; 2000; 2002; 2006-pres |
| Chile | 1987-2011 | | 2012-present |
| Croatia | 1992-2007; 2016 | | 2008-2015; 2017-present |
| Cyprus | 1987 | | 1988-present |
| Czech Republic | 1992-2005 | | 2006-present |
| Equatorial Guinea | 1987-2006; 2015-present | | 2007-2014 |
| Estonia | 1991-2005 | | 2006-present |
| Guam | 1990-1994 | | 1987-1989; 1995-present |
| Greece | 1987-1995 | | 1996-present |
| Hungary | 1987-2006; 2012-2013 | | 2007-2011; 2014-present |
| Isle of Man | 1990-2001 | | 1987-1989; 2002-present |
| Latvia | 1991-2008; 2010-2011 | | 2009; 2012-present |
| Lithuania | 1991-2011 | | 2012-present |
| Macao (SAR) | 1987-1993 | | 1994-present |
| Malta | 1987-1988; 1990-1997;1999; 2001 | | 1989; 1998; 2000; 2002-pres. |
| Mauritius | 1987-2018; 2020-present | | 2019 |
| Nauru | 2016-2018 | | 2015; 2019-present |
| New Caledonia | 1987-1994 | | 1995-present |
| Northern Mariana Islands | 1992-1994; 2002-2006 | | 1995-2001; 2007-present |
| Oman | 1987-2006 | | 2007-present |
| Palau | 1987-2015 | | 2016-present |
| Panama | 1987-2016; 2020-present | | 2017-2019 |
| Poland | 1987-2008 | | 2009-present |
| Portugal | 1987-1993 | | 1994-present |
| Puerto Rico | 1987-1988; 1990-2001 | | 1989; 2002-present |
| Republic of Korea | 1987-1994; 1998-2000 | | 1995-1997; 2001-present |
| Romania | 1987-2018; 2020-present | | 2019 |
| Russia | 1991-2011; 2015-present | | 2012-2014 |
| Seychelles | 1987-2013 | | 2014-present |
| Slovak Republic | 1992-2006 | | 2007-present |
| Slovenia | 1992-1996 | | 1997-present |
| Saudi Arabia | 1990-2003 | | 1987-1989; 2004-present |
| St. Kitts and Nevis | 1987-2010 | | 2011-present |
| Trinidad and Tobago | 1987-2005 | | 2006-present |
| Uruguay | 1987-2011 | | 2012-present |
| Venezuela | 1987-2013; 2015-present | | 2014 |

| **High-income countries** | | | |
| --- | --- | --- | --- |
| Andorra | Faeroe Islands | Kuwait | St. Martin- French |
| Australia | Finland | Liechtenstein | Sweden |
| Austria | France | Luxembourg | Switzerland |
| Bahamas | French Polynesia | Monaco | Taiwan |
| Belgium | Germany | Netherlands | Turks & Caicos Isl |
| Bermuda | Greenland | New Zealand | Untd Arab Emir. |
| Brunei Darussalam | Hong Kong (SAR) | Norway | United Kingdom |
| Canada | Iceland | Qatar | United States |
| Cayman Islands | Ireland | San Marino | Virgin Islands- US |
| Channel Islands | Israel | Singapore |  |
| Curacao | Italy | St. Martin (Dutch) |  |
| Denmark | Japan | Spain |  |

# **Appendix 3. Details of search strategy**

To identify relevant studies for our review, we have developed a set of English search terms and a search strategy in collaboration with an information specialist. Our search terms combine Boolean terms with a list of keywords related to the review's inclusion criteria, which we will use to search electronic databases and institutional websites with sufficient search functionality. The search strategy was initially designed for and tested in the Web of Science Core collection.^[[1]](#footnote-2)^ Due to time limitations, the search string did not undergo formal peer review by a second information specialist, but it was reviewed by other members of the core research team.

| Farms | 1 | TS=(farm* OR agricultur* OR "smallhold*" OR "small hold*" OR microfarm* OR "micro-farm*" OR pastoral* OR agropastoral OR "agro-pastoral" OR ejido OR silvopastoral OR eco-agricultur* OR "market garden*" OR "producer*" OR "grower*" OR "agronomy" OR "husbandry" OR "floricultur*" OR "horticultur*" OR polyculture OR "livestock" OR "crop" OR "crops" OR "outgrower*") |
| --- | --- | --- |
| Infrastructure interventions | 2 | TS = (transport* OR road* OR bridge* OR waterway* OR boat* OR rail* OR "train tracks" OR ports OR airport* OR "border crossing*" OR "distribution facilit*" OR highway* OR ((distribut* OR freight OR access* OR connect* OR rural OR electric* OR power OR transmission OR grid OR energy) NEAR/3 (infrastructure OR expansion OR expand*))) |
| Output market information | 3 | TS=("information dissemination" OR "communication technolog*" OR "information technolog*" OR "cellphone*" OR "cell phone*" OR SMS OR "text messag*" OR "information service*" OR "internet" OR "website*" OR "app" OR "apps" OR "mobile device*" OR "mobile phone*" OR "mobile telephon*" OR "market information" OR "price information" OR "telecommunicat*" OR "televis*" OR "radio*" OR "social network*" OR "peer network*" OR "information campaign*" OR ((communication OR information OR dissemination OR data OR initiative* OR diffusion OR diffusion) NEAR/3 (technolog* OR infrastructur*))) |
| New and alternative marketplaces and marketing | 4 | TS= ((online OR internet OR website OR app OR apps OR mobile) NEAR/3 ("commodity exchange" OR market*)) OR TS=(("trader*" OR "processor*" OR "agro-exporter*" OR "agroexporter*" OR "dealer*" OR "agro-dealer*" OR "agrodealer*" OR "wholesaler*" OR "miller*" OR "village merchant*" OR "commission agent*" OR "broker*" OR "retailer*" OR "aggregator*" OR "buyer*" OR "trading firm*" OR "farm-firm*" OR "tender*" OR "service provider*" OR "trucker*" OR "transporter*" OR "logistics enterprise*" OR "third-party logistics" OR "freight forwarder*" OR "food manufacturer*" OR "packager*" OR "distributor*") NEAR/3 (commit* OR agree* OR contract* OR "fixed pric*" OR "arrang*" OR "guarant*" OR "advanc*")) OR TS=("market modernization" OR "market modernisation" OR "modern market*" OR "innovative marketing channel*" OR "new marketing channel*" OR commercialization OR commercialisation OR "high-value chain*" OR "high-value market*" OR "high value agricultur*" OR "agrifood transformation*" OR "agri-food transformation*" OR "e-commerce" OR "market reform*" OR "market linkage*" OR "online agricultural platform*" OR "agri-platform*" OR agriplatform* OR "market hub*" OR "internet kiosk*") OR TS=(("vertical") NEAR/2 ("integration" OR "coordination" OR "linkage*")) |
| Contract farming | 5 | TS=(contract* OR "nucleus estate*" OR "outgrower*" OR "out grower*") |
| Storage infrastructure | 6 | TS=((storage OR storing OR stored) NEAR/5 (innovat* OR infrastructure)) OR TS=("warehous*" OR "shed" OR "sheds" OR "satellite collection point*") |
| Intervention terms | 7 | #2 OR #3 OR #4 OR #5 OR #6 |
| LMICS^[[2]](#footnote-3)^ | 8 | TS=(afghanistan or albania or algeria or "american samoa" or angola or "antigua and barbuda" or antigua or barbuda or argentina or armenia or armenian or aruba or azerbaijan or bahrain or bangladesh or barbados or "republic of belarus" or belarus or byelarus or belorussia or byelorussian or belize or "british honduras" or benin or dahomey or bhutan or bolivia or bosnia or herzegovina or botswana or bechuanaland or brazil or brasil or bulgaria or "burkina faso" or "burkina fasso" or "upper volta" or burundi or urundi or "cabo verde" or "cape verde" or cambodia or kampuchea or "khmer republic" or cameroon or cameron or cameroun or "central african republic" or "ubangi shari" or chad or chile or china or colombia or comoros or "comoro islands" or "iles comores" or mayotte or congo or zaire or "costa rica" or "cote d’ivoire" or "cote d’ ivoire" or "cote divoire" or "cote d ivoire" or ivory coast or croatia or cuba or cyprus or "czech republic" or czechoslovakia or djibouti or "french somaliland" or dominica or "dominican republic" or ecuador or egypt or "united arab republic" or "el salvador" or "equatorial guinea" or "spanish guinea" or eritrea or estonia or eswatini or swaziland or ethiopia or fiji or gabon or "gabonese republic" or gambia or "georgia (republic)" or georgian or ghana or "gold coast" or gibraltar or greece or grenada or guam or guatemala or guinea or "guinea bissau" or guyana or "british guiana" or haiti or hispaniola or honduras or hungary or india or indonesia or timor or iran or iraq or "isle of man" or jamaica or jordan or kazakhstan or kazakh or kenya or korea or kosovo or kyrgyzstan or kirghizia or kirgizstan or "kyrgyz republic" or kirghiz or laos or "lao pdr" or "lao people's democratic republic" or latvia or lebanon or "lebanese republic" or lesotho or basutoland or liberia or libya or "libyan arab jamahiriya" or lithuania or macau or macao or macedonia or madagascar or "malagasy republic" or malawi or nyasaland or malaysia or "malay federation" or "malaya federation" or maldives or "indian ocean" or mali or malta or micronesia or kiribati or "marshall islands" or nauru or "northern mariana islands" or palau or tuvalu or mauritania or mauritius or mexico or moldova or moldovian or mongolia or montenegro or morocco or ifni or mozambique or "portuguese east africa" or myanmar or burma or namibia or nepal or "netherlands antilles" or nicaragua or niger or nigeria or oman or muscat or pakistan or panama or "new guinea" or paraguay or peru or philippines or philipines or phillipines or phillippines or poland or "polish people's republic" or portugal or "portuguese republic" or "puerto rico" or romania or russia or "russian federation" or ussr or "soviet union" or "union of soviet socialist republics" or rwanda or ruanda or samoa or "pacific islands" or polynesia or "samoan islands" or "navigator island" or "navigator islands" or "sao tome and principe" or "saudi arabia" or senegal or serbia or seychelles or "sierra leone" or slovakia or "slovak republic" or slovenia or melanesia or "solomon island" or "solomon islands" or "norfolk island" or "norfolk islands" or somalia or "south africa" or "sri lanka" or ceylon or "saint kitts and nevis" or "st. kitts and nevis" or "saint lucia" or "st. lucia" or "saint vincent and the grenadines" or "saint vincent" or "st. vincent" or grenadines or sudan or suriname or surinam or "dutch guiana" or "netherlands guiana" or syria or "syrian arab republic" or tajikistan or tadjikistan or tadzhikistan or tadzhik or tanzania or tanganyika or thailand or siam or "timor leste" or "east timor" or togo or "togolese republic" or tonga or trinidad or tobago or tunisia or turkey or turkmenistan or turkmen or uganda or ukraine or uruguay or uzbekistan or uzbek or vanuatu or "new hebrides" or venezuela or vietnam or "viet nam" or "middle east" or "west bank" or gaza or palestine or yemen or yugoslavia or zambia or zimbabwe or "northern rhodesia" or "global south" or africa or magreb or maghrib or sahara or "west indies" or "indian ocean islands" or caribbean or "central america" or "latin america" or "south and central america" or "south america" or "asia, central" or "central asia" or "asia, northern" or "north asia" or "northern asia" or "asia, southeastern" or "southeastern asia" or "south eastern asia" or "southeast asia" or "south east asia" or "asia, western" or "western asia" or "europe, eastern" or "east europe" or "eastern europe" or "developing country" or "developing countries" or "developing nation$" or "developing population$" or "developing world" or "less developed countr*" or "less developed nation$" or "less developed population$" or "less developed world" or "lesser developed countr*" or "lesser developed nation$" or "lesser developed population$" or "lesser developed world" or "under developed countr*" or "under developed nation$" or "under developed population$" or "under developed world" or "underdeveloped countr*" or "underdeveloped nation$" or "underdeveloped population$" or "underdeveloped world" or "middle income countr*" or "middle income nation$" or "middle income population$" or "low income countr*" or "low income nation$" or "low income population$" or "lower income countr*" or "lower income nation$" or "lower income population$" or "underserved countr*" or "underserved nation$" or "underserved population$" or "underserved world" or "under served countr*" or "under served nation$" or "under served population$" or "under served world" or "deprived countr*" or "deprived nation$" or "deprived population$" or "deprived world" or "poor countr*" or "poor nation$" or "poor population$" or "poor world" or "poorer countr*" or "poorer nation$" or "poorer population$ or poorer world or developing econom* or less developed econom* or lesser developed econom*" or "under developed econom*" or "underdeveloped econom*" or "middle income econom*" or "low income econom*" or "lower income econom*" or "low gdp" or "low gnp" or "low gross domestic" or "low gross national" or "lower gdp" or "lower gnp" or "lower gross domestic" or "lower gross national" or lmic or lmics or "third world" or "lami countr*" or "transitional countr*" or "emerging economies" or "emerging nation$") |
| LMICs^[[3]](#footnote-4)^ | 9 | TS=(afghan or afghans or afghani or albanian$ or algerian$ or "american samoan$" or angolan$ or antiguan$ or barbudan$ or argentine$ or argentinian$ or argentinean$ or armenian$ or aruban$ or azerbaijani$ or bahraini$ or bangladeshi$ or bangalees or bajan$ or belarusian$ or byelorussian$ or belizean$ or beninese$ or bhutanese or bolivian$ or bosnian$ or botswana or batswana or brazilian$ or brasilian$ or bulgarian$ or burkinabe or burkinese or burundian$ or "cape verdean$" or "cabo verdean$" or cambodian$ or khmer or cameroonian$ or "central african$" or chadian$ or chilean$ or chinese or colombian$ or comorian$ or congolese or "costa rican$" or ivorian$ or croatian$ or cuban$ or cypriot$ or czech$ or djiboutian$ or dominican$ or ecuadorian$ or egyptian$ or salvadoran$ or "equatorial guinean$" or equatoguinean$ or eritrean$ or estonian$ or swazi$ or swati$ or ethiopian$ or fijian or gabonese or gabonaise or gambian$ or georgian$ or ghanaian$ or gibraltarian$ or greek$ or grenadian$ or guamanian$ or guatemalan$ or guinean$ or "bissau guinean$" or guyanese or haitian$ or honduran$ or hungarian$ or indian$ or indonesian$ or iranian$ or iraqian$ or iraqi$ or manx or jamaican$ or jordanian$ or kazakhstani$ or kenyan$ or kirabati or kirabatian$ or "north korean$" or korean$ or kosovar$ or kosovan$ or kyrgyz* or lao or laotian$ or latvian$ or lebanese or lesothan$ or lesothonian$ or mosotho or basotho or liberian$ or libyan$ or lithuanian$ or macanese or macedonian$ or malagasy or madagascan$ or malawian$ or malaysian$ or maldivian$ or malian$ or maltese or marshallese$ or mauritanian$ or mauritian$ or mexican$ or micronesian$ or moldovan$ or mongolian$ or mongol or montenegrin$ or moroccan$ or mozambican$ or burmese or myanma or namibian$ or nauruan$ or nepali or nepalese or "netherlands antillean$" or nicaraguan$ or nigerien$ or nigerian$ or "northern mariana islander$" or mariana$ or omani$ or pakistani$ or palauan$ or panamanian$ or "papua new guinean$" or paraguayan$ or peruvian$ or philippine$ or philipine$ or phillipine$ or phillippine$ or filipino$ or filipina$ or polish or pole or poles or portuguese or "puerto rican$" or romanian$ or russian$ or "soviet people" or "soviet population" or rwandan$ or rwandese or ruandan$ or ruandese or samoan$ or "sao tomean$" or santomean$ or "saudi arabian$" or saudi$ or senegalese or serbian$ or montenegrin$ or seychellois or seychelloise$ or "sierra leonean$" or slovak$ or slovene$ or "solomon islander$" or somali$ or "south african$" or "south sudanese" or "sri lankan$" or ceylonese or kittitian$ or nevisian$ or "saint lucian$" or vincentian$ or sudanese or surinamese$ or syrian$ or tajik$ or tajikistani$ or tanzanian$ or tanganyikan$ or thai or timorese$ or togolese or tongan$ or trinidadian$ or tobagonian$ or tunisian$ or turk$ or turkish or turkmen$ or tuvaluan$ or ugandan$ or ukrainian$ or uruguayan$ or uzbek$ or vanuatu* or venezuelan$ or vietnamese or yemeni$ or yemenite$ or yemenese or yugoslav$ or yugoslavian$ or zambian$ or zimbabwean$ or african$ or asian$ or "pacific islander$" or "latin american$" or "central american$" or "south american$" or caribbean$ or "west indian$" or iberoamerican$ or "middle eastern" or "middle eastern*") |
| LMICs | 10 | #8 OR #9 |
| Study design | 11 | TS=((match* NEAR/2 (propensity or coarsened or covariate or neighbo$r)) or "propensity score" or ("difference* in difference*" or "difference-in-difference*" or "differences-in-difference*" or "double difference*") or (quasi-experiment* or "quasi experiment*") or (estimator and evaluat*) or ("instrumental variable*" or (IV NEAR/2 (estimation or approach))) or (Heckman NEAR/3 (model* or approach*)) or ((two-stage or "two stage") NEAR/3 (control* or function* or "least squares")) or "regression discontinuity" or "time series" or counterfactual or "segment* regression" or (non NEAR/2 participant*) or ((control or comparison) NEAR/2 (group* or condition* or area* or village* or household* or intervention)) or (panel* NEAR/2 (data or household* or model*)) or ((exploit* or "tak* advantage") NEAR/3 (variation* or variety or exogen* or heterogen*)) or (econometric NEAR/2 (model* or adjust*)) or (select* NEAR/2 (bias* or self))) |
| Study design | 12 | TS=((experiment* NEAR/2 (design or study or research or evaluation or evidence or vary or varies or variation)) or ((random or randomi?ed or randomly) NEAR/2 (trial or assign* or treatment or control* or allocat* or experiment* or vary or varies or variation or choose or chose*))) |
| Study design | 13 | TS=((impact$ or effect*) NEAR/2 (evaluat* or assess or assessing or assessment or analyze or analyse or analyzing or analysing or analysis or analytical or estimate or estimating or estimation or cause or causal)) |
| Study design | 14 | TS=("program* evaluation" or "project evaluation" or "evaluation research" or "natural experiment*" or "program* effectiveness" or "outcome assessment" or "evaluation study" or "field experiment") |
| All study design | 15 | #11 OR #12 OR #13 OR #14 |
| All Combined | 16 | #1 AND #7 AND #10 AND #15 |
| Restrict to appropriate sub-databases |  | Social Sciences Citation Index (SSCI), Science Citation Index Expanded (SCI-EXPANDED), Conference Proceedings Citation Index – Science (CPCI-S), Conference Proceedings Citation Index – Social Science & Humanities (CPCI-SSH), Emerging Sources Citation Index (ESCI) |
| Publication year |  | Limit publication year 2000-01-01 to present |

# **Appendix 4. Details of process of search strategy development**

A combination of techniques and resources informed the final search strategy used to search the sources of bibliographic information listed in Table 5. The search strategy was composed of search strings aimed to retrieve literature describing farm- and agriculture-related output market access interventions as outlined in Table 2. In addition, search strings routinely applied and previously tested in systematic reviews by 3ie on the effects of interventions were used to limit the search results to experimental and quasi-experimental study designs in low- and middle-income countries.

*Identifying a sample of studies for developing the search strategy*

To develop our search strategy and perform pearl-harvesting techniques, we identified an initial sample of relevant studies by consulting with members of the project’s expert advisory group and searching the results of a selection of existing reviews (see Appendix 5 below). We also performed targeted searches of 3ie’s Development Evidence Portal (DEP), which is currently the world’s most expansive repository of rigorous evidence on what works in international development. At the time of this writing, it includes 10,795 impact evaluations and 990 systematic reviews, of which 1,084 impact evaluations and 55 systematic reviews are from the agriculture, fishing, and forestry sector.

**Table of search filters used for a targeted search of 3ie’s DEP to identify an initial sample of studies for search strategy development**

| **Intervention type** | **Search filters** |
| --- | --- |
| Farm to market interventions | "Transportation” |
| Output market information interventions | "Agricultural market information"  "Radio messages for agricultural production"  "Voice messages for agricultural production"  "Mobile video messages for agricultural production"  "Text/SMS messages for agricultural production" |
| Initiatives creating new marketplaces & alternative marketing opportunities | "Advance agricultural sales commitments"  "Access to output markets for smallholder farmers"  "Access to output markets for farmer-based organizations" |
| Contract farming | "Organisational contract farming (agriculture)" |
| Improved storage infrastructure and technologies | "Storage credit for agricultural products" "Warehousing and other storage services for agricultural products" |

Notes: Search filters are mapped approximately to the intervention type we expect they are most relevant.

We used the DEP’s advanced search filters to perform targeted searches of the DEP. The search filters are based on the portal’s intervention taxonomy, which indexes studies using a standardised vocabulary for classifying interventions (see Kozakiewicz et al, 2021). We identified 248 studies using the intervention filters in the table above, which we screened for relevance. Appendix 6 provides a list of the initial list of the 100 studies we provisionally identified for peal-harvesting and developing our search strategy.

*Process for developing search terms*

An initial search strategy was developed based on intervention terms and descriptions in the draft protocol, terms gathered manually from the titles and abstracts of the initial list of studies described above, and search strings adapted from previously published reviews on related topics and 3ie’s standard search . The search was then further enhanced using two text analysis methods: a word frequency and word analysis of the initial list of studies previously identified and term harvesting with the R package *litsearchr* on a subset of the initial list. *litsearchr* uses a statistical measure based on keyword co-occurrence to identify important terms in a set of titles and abstracts (Grames et al., 2019). The terms suggested by these two methods were reviewed by the information specialist, and if not already captured by the search and likely to add relevant studies with minimal noise, were added to the search strategy.

Finally, the search was iteratively tested against a subset of the initially identified studies (n=55; i.e. those indexed in Web of Science Core Collection databases) to determine its sensitivity to finding relevant studies. Those that were not found were examined for potentially missing keywords that could be added to the search. The final search strategy was able to retrieve 42 (76%) of the 55 initial studies identified and indexed in Web of Science. Studies not found were most typically missing terminology included in the study design search string. This can be considered a limitation of the search strategy in its ability to comprehensively retrieve all potentially eligible studies. However, supplemental methods such as forward and backward citation tracking, and other grey literature searches, should mitigate this limitation to some extent.

# **Appendix 5. List of related evidence maps and reviews**

Aker, JC, Ghosh, I and Burrell, J, 2016. The promise (and pitfalls) of ICT for agriculture initiatives. *Agricultural Economics,* 47(S1), pp.35–48.

Aker, JC and Mbiti IM, 2010. Mobile Phones and Economic Development in Africa. *Journal of Economic Perspectives,* 24(3), pp.207–32.

Bellemare, MF and Bloem, JR, 2018. Does contract farming improve welfare? A review. *World development,* 112, pp.259-271.

Daccache, A, Knox, J and Hess, T, 2013. *What is the impact of infrastructural investments in roads, electricity and irrigation on agricultural productivity?* Final review. Bangor, UK: Collaboration for Environmental Evidence (CEE). Available at: https://assets.publishing.service.gov.uk/media/57a08a0840f0b652dd00051a/CEE11-007_SystematicReview.pdf

Dillon, B and Dambro, C, 2017. How Competitive Are Crop Markets in Sub-Saharan Africa? *American Journal of Agricultural Economics,* 99(5), pp.1344–1361.

Hainzer, K, Best, T and Brown, PH, 2019. Local value chain interventions: a systematic review. *Journal of Agribusiness in Developing and Emerging Economies,* 9(4), pp.369–390.

Hine, J, Abedin, M, Stevens, RJ, Airey, T and Anderson, T, 2016. *Does the extension of the rural road network have a positive impact on poverty reduction and resilience for the rural areas served? If so how, and if not why not?* A systematic review. London: EPPI-Centre, Social Science Research Unit, UCL Institute of Education, University College London.

Hine, J, Sasidharan, M, Torbaghan, ME, Burrow, M and Usman, K, 2019. *Evidence of the impact of rural road investment on poverty reduction and economic development,* Knowledge, Evidence & Learning for Development (K4D) Helpdesk Report. Brighton, UK: Institute of Development Studies.

Lopez-Avila, D, Husain, S, Bhatia, R, Nath, M, and Vinaygyam, R. 2017. Agricultural innovation: an evidence gap map, 3ie Evidence Gap Map Report 12. New Delhi: International Initiative for Impact Evaluation (3ie).

Ludwig, C, Nagarajan, G and Zaman, L, 2016. *Systematic Review of the Effects of Rural Roads on Expanding Agricultural Markets in Developing Countries*. Arlington, VA: Social Impact, Inc.

Miller, DC, Ordoñez, PJ, Brown, SE, Forrest, S, Nava, NJ, Hughes, K and Baylis, K, 2020. The impacts of agroforestry on agricultural productivity, ecosystem services, and human well-being in low-and middle-income countries: An evidence and gap map. *Campbell Systematic Reviews,* 16(1), e1066.

Moore, N., Glandon, D., Tripney, J., Kozakiewicz, T., Shisler, S., Eyers, J., Zalfou, R., Leon, M.D.A., Kurkjian, V., Snilstveit, B. and Perdana, A. 2020. Effects of electricity access interventions on socio-economic outcomes in low- and middle-income countries. 3ie Systematic Review 45. London: International Initiative for Impact Evaluation (3ie).

Moore, N, Lane, C, Storhaug, I, Franich, A, Rolker, H, Furgeson, J, Sparling, T and Snilstveit, B. 2021. The effects of food systems interventions on food security and nutrition outcomes in low- and middle-income countries, 3ie Evidence Gap Map Report 16. New Delhi: International Initiative for Impact Evaluation (3ie).

Nakasone, E, Torero, M and Minten, B, 2014. The power of information: The ICT revolution in agricultural development. *Annual Review of Resource Economics,* 6(1), pp.533–550.

Nandi, R, Nedumaran, S and Ravula, P, 2021. The interplay between food market access and farm household dietary diversity in low and middle income countries: A systematic review of literature. *Global Food Security,* 28, 100484.

Neza, K, Nyarko, Y and Orozco, A, 2021. *Digital Trading and Market Platforms: Ghana Case Study* in Madon T, Anderson RJ, Casaburi L, Lee K, Rezaee A and Gadgil, AJ (eds.), An Introduction to Development Engineering, Springer.

Nguyen, AT, Dzator, J and Nadolny, A, 2015. Does contract farming improve productivity and income of farmers?: A review of theory and evidence. *The Journal of Developing Areas,* 49(6), pp.531–538.

Policy and Operations Evaluation Department (IOB), 2011. *Improving Food Security: A Systematic Review of the Impact of Interventions in Agricultural Production, Value Chains, Market Regulation, and Land Security.* Den Haag: Policy and Operations Evaluation Department (IOB), Ministry of Foreign Affairs.

Ton, G, Desiere, S, Vellema, W, Weituschat, S and D'Haese, M, 2017. The effectiveness of contract farming for raising income of smallholder farmers in low- and middle-income countries: a systematic review. *Campbell Systematic Reviews,* 13(1), pp.1–131.

# **Appendix 6. The initial sample of studies used for search strategy development**

***Farm to market interventions (n=25)***

Ali, R, Barra, AF, Berg, CN, Damania, R, Nash, J and Russ, J, 2015. *Transport Infrastructure and Welfare: An Application to Nigeria,* Policy Research Working Paper 7271. Washington, DC: World Bank.

Asher, S and Novosad, P, 2020. Rural Roads and Local Economic Development. *American Economic Review,* 110(3), pp.797–823.

Blankespoor, B, Emran, MS, Shilpi, F and Xu, L, 2018. *Bridge to Bigpush or Backwash? Market Integration, Reallocation, and Productivity Effects of Jamuna Bridge in Bangladesh,* SSRN Scholarly Paper 3162451. Rochester, NY: Social Science Research Network.

Bonilla, J, McCarthy, N, Mugatha, SM, Rai, N, Coombes, A and Brubaker, J, 2018. *Impact evaluation of the smallholder dairy commercialization programme in Kenya,* Impact Evaluation Report 73. New Delhi: International Initiative for Impact Evaluation (3ie).

Brooks, W and Donovan, K, 2020. Eliminating uncertainty in market access: The impact of new bridges in rural Nicaragua. *Econometrica,* 88(5), pp.1965–1997.

Casaburi, L, Glennerster, R and Suri, T, 2013. *Rural roads and intermediated trade: Regression discontinuity evidence from Sierra Leone,* SSRN Scholarly Paper 2161643. Rochester, NY: Social Science Research Network.

Charlery, LC, Qaim, M and Smith-Hall, C, 2016. Impact of infrastructure on rural household income and inequality in Nepal. *Journal of Development Effectiveness,* 8(2), pp.266–286.

Cook, C, Duncan, T, Jitsuchon, S, Sharma, A and Guobao, W, 2004. *Assessing the Impact of Transport and Energy Infrastructure on Poverty Reduction. Manila: ADB.* Mandaluyong City: Asian Development Bank (ADB).

Dercon, S and Hoddinott, J, 2005. *Livelihoods, growth, and links to market towns in 15 Ethiopian villages*. FCND Discussion Paper 194. Washington, DC: International Food Policy Research Institute.

Dorosh, P, Wang, HG, You, L and Schmidt, E, 2010. *Crop production and road connectivity in sub-Saharan Africa: a spatial analysis.* Policy Research Working Paper 5385. Washington, DC: World Bank.

Dumas, C and Játiva, X, 2020. *Better roads, better off?: Evidence on improving roads in Tanzania,* FSES Working Papers 518. Fribourg, Switzerland: Faculty of Economics and Social Sciences, University of Freiburg.

Khandker, SR and Koolwal, GB, 2011. *Estimating the long-term impacts of rural roads: a dynamic panel approach.* Policy Research Paper 5867. Washington, DC: World Bank.

Khandker, SR and Koolwal, GB, 2010. How Infrastructure and Financial Institutions Affect Rural Income and Poverty: Evidence from Bangladesh. *Journal of Development Studies,* 46(6), pp.1109–1137.

Khandker, SR, Bakht, Z and Koolwal, GB, 2009. The Poverty Impact of Rural Roads: Evidence from Bangladesh. *Economic Development and Cultural Change,* 57(4), pp.685–722.

Kingombe, CKM, di Falco, S, 2012. *The impact of a feeder road project on cash crop production in Zambia’s Eastern Province between 1997 and 2002*. Paper presented at the CSAE Conference, held on March, Oxford.

Limi, A. and Smith, J, 2007. *What is missing between agricultural growth and infrastructure development? cases of coffee and dairy in Africa*, Policy Research working paper 4411. Washington, DC. World Bank.

Lin, J, Zhang, Z, Liu, Z and Rommel, J, 2020. The impact of cooperatives’ transportation services on farm income: Evidence from tobacco farmers in Guizhou, China. *Agribusiness,* 36(1), pp.146–158.

Mu, R., van de Walle, D, 2007. *Rural roads and local market development in Vietnam,* Policy Research Working Paper 4340. Washington, DC: World Bank.

Nakamura, S, Bundervoet, T and Nuru, M, 2020. Rural roads, poverty, and resilience: Evidence from Ethiopia. *The Journal of Development Studies,* 56(10), pp.1838–1855.

Qin, Y and Zhang, X, 2016. *The Road to Specialization in Agricultural Production: Evidence from Rural China*. *World Development,* pp.1–17.

Ruijs, A, Schweigman, C, Lutz, C, 2004. The impact of transport– and transaction-cost reductions on food markets in developing countries: evidence for tempered expectations for Burkina Faso*. Agricultural Economics,* 31, pp.219–228.

Shamdasani, Y, 2017. *Essays on Agricultural and Labor Markets in India*. Doctoral thesis, Columbia University.

Shamdasani, Y, 2021. Rural road infrastructure & agricultural production: Evidence from India. *Journal of Development Economics,* 152, 102686.

Stifel, D, Minten, B, Dorosh, P, 2003. *Transaction costs and agricultural productivity: implications of isolation for rural poverty in Madagascar,* MSSD Discussion Paper No. 56. Washington, DC: International Food Policy Research Institute.

Wondemu, KA, 2010. *Road infrastructure and rural poverty in Ethiopia,* Doctoral thesis. Development and Economic Studies Department, University of Bedford.

***Output market information interventions (n=17)***

Abate, GT, Tanguy B, de Brauw, A and Minot N, 2018. The Impact of the Use of New Technologies on Farmers’ Wheat Yield in Ethiopia: Evidence from a Randomized Control Trial. *Agricultural Economics*, 49 (4), pp.409–21.

Aker, JC, 2008. *Does Digital Divide or Provide The Impact of Mobile Phones on Grain Markets in Niger*. Center for Global Development Working Paper No 154.

Camacho, A and Conover, E, 2011. *The impact of receiving price and climate information in the agricultural sector,* IDB working paper 220. Washington, DC: Inter-American Development Bank (IDB).

Cook, C, Duncan, T, Jitsuchon, S, Sharma, A and Guobao, W, 2004. *Assessing the Impact of Transport and Energy Infrastructure on Poverty Reduction. Manila: ADB.* Mandaluyong City: Asian Development Bank (ADB).

Courtois, P and Subervie. J, 2015. Farmer Bargaining Power and Market Information Services. *American Journal of Agricultural Economics,* 97(3), pp.953–77.

Fafchamps, M and Minten, B, 2012. Impact of SMS-Based Agricultural Information on Indian Farmers. *The World Bank Economic Review,* 26(3), pp.383–414.

Hildebrandt, N, Nyarko, Y, Romagnoli, G and Soldani, E, 2020. *Price Information, Inter-Village Networks, and “Bargaining Spillovers”: Experimental Evidence from Ghana*, SSRN Scholarly Paper 3694558. Rochester, NY: Social Science Research Network.

Khandker, SR and Koolwal, GB, 2010. How Infrastructure and Financial Institutions Affect Rural Income and Poverty: Evidence from Bangladesh. *Journal of Development Studies,* 46(6), pp.1109–1137.

Koirala, B, Bohara, A, Devkota, S and Upadhyaya, K. 2019. Community managed hydropower, spillover effect and agricultural productivity: The case of rural Nepal. *World Development Perspectives,* 13, pp.67–74.

Limi, A. and Smith, J, 2007. *What is missing between agricultural growth and infrastructure development? cases of coffee and dairy in Africa*, Policy Research working paper 4411. Washington, DC. World Bank.

Mitra, S, Mookherjee, D, Torero, M and Visaria, S, 2018. Asymmetric Information and Middleman Margins: An Experiment with Indian Potato Farmers. *The Review of Economics and Statistics*, 100(1), pp.1–13.

Nakasone, E, 2013. *The role of price information in agricultural markets: experimental evidence from rural Peru*. Paper presented at the Agricultural & Applied Economics Association’s 2013 AAEA & CAES Joint Annual Meeting, held on the 4-6 August 2013, Washington, DC.

Ogutu, SO, Okello, JJ and Otieno, DJ, 2014. Impact of Information and Communication Technology-Based Market Information Services on Smallholder Farm Input Use and Productivity: The Case of Kenya. *World Development,* 64(December), pp.311–21.

Svensson, J and Yanagizawa, D, 2009. Getting Prices Right: The Impact of the Market Information Service in Uganda. *Journal of the European Economic Association,* 7(2/3), pp.435–445.

Tack, J and Aker, J, 2014. Information, Mobile Telephony, and Traders’ Search Behavior in Niger. *American Journal of Agricultural Economics,* 96(5), pp.1439–54.

Yanagizawa-Drott, D and Svensson, J, 2012. *Estimating Impact in Partial vs. General Equilibrium: A Cautionary Tale from a Natural Experiment in Uganda*. Cambridge, MA: John F. Kennedy School of Government at Harvard University.

Yang, M. 2003. China's rural electrification and poverty reduction. *Energy Policy,* 31(3), pp.283–295.

***New marketplaces and alternative marketing opportunities (n=7)***

Ashraf, N, Giné, X and Karlan, D, 2009. Finding Missing Markets (and a Disturbing Epilogue): Evidence from an Export Crop Adoption and Marketing Intervention in Kenya. *American Journal of Agricultural Economics,* 91(4), pp.973–90.

Bayiyana, I, Hepelwa, A, Rao, EJO and Mdadila, K, 2018. Do Dairy Market Hubs Improve Smallholder Farmers’ Income? The Case of Dairy Farmers in the Tanga and Morogoro Regions of Tanzania. *Agrekon,* 57(2), pp.121–36.

Bold, T, Ghisolfi, S, Nsonzi, F and Svensson, J, (in press). Market Access and Quality Upgrading: Evidence from Four Field Experiments. *American Economic Review*.

Bergquist, FL and McIntosh, C, 2021. *Search Cost, Intermediation, and Trade: Experimental Evidence from Ugandan Agricultural Markets,* CEGA Working Papers WPS 173. Berkeley, California: Center for Effective Global Action (CEGA), University of California, Berkeley.

Goyal, A, 2010. Information, Direct Access to Farmers, and Rural Market Performance in Central India. *American Economic Journal: Applied Economics,* 2(3), pp.22–45.

 Levi, R, Rajan, M, Singhvi, S and Zheng, Y, 2020. The Impact of Unifying Agricultural Wholesale Markets on Prices and Farmers’ Profitability. *Proceedings of the National Academy of Sciences,* 117(5), pp.2366–71.

Maertens, A, Mhango, W and Michelson, H, 2020. *The effect of demonstration plots and the warehouse receipt system on integrated soil fertility management adoption, yield and income of smallholder farmers: a study from Malawi’s Anchor Farms,* 3ie Impact Evaluation Report 122. New Delhi: International Initiative for Impact Evaluation (3ie).

***Contract farming*** ***(n=47)***

Adebisi, LO, Jimoh, O, Asuquo, J, Osasona, KK and Ojediran, EO, 2019. Effect of Contract Farming on Poultry Farming Households Food Security in Osun State, Nigeria*. Agro-Science,* 18(1), pp.45–49.

Arouna, A, Michler, JD and Lokossou, JC, 2019. *Contract Farming and Rural Transformation: Evidence from a Field Experiment in Benin,* 25665: National Bureau of Economic Research.

Awotide, BA., Fashogbon A and Awoyemi, TT, 2015. *Impact of Agro-Industrial Development Strategies on Smallholder Rice Farmers’ Productivity, Income and Poverty: The Case of Contract Farming in Nigeria.* Presented at the International Conference of the Centre for the Studies of African Economies (CSAE), held on the 22-24 March 2015, St. Catherine’s College, Oxford, United Kingdom.

Balineau, G, 2013. Disentangling the effects of fair trade on the quality of Malian cotton. *World Development,* 44, pp.241–255.

Bannor, RK., Oppong-Kyeremeh, H, and Adjei-Addo, E, 2017. Improving the income of small scale rice producers through outgrower scheme in the Volta Region of Ghana*. Indian Journal of Economics and Development,* 13(2), pp.584–590.

Bellemare, MF, 2012. As You Sow, So Shall You Reap: The Welfare Impacts of Contract Farming. *World Development,* 40(7), pp.1418–34.

Bellemare, MF, Lee, YN, and Novak, L, 2017. *Contract farming as partial insurance*, Working Paper. Minnesota: University of Minnesota.

Bezabeh, A, Beyene, F, Haji, J and Lemma, T, 2020. Impact of Contract Farming on Income of Smallholder Malt Barley Farmers in Arsi and West Arsi Zones of Oromia Region, Ethiopia. *Cogent Food & Agriculture,* 6(1), 1834662.

Bolwig, S, Gibbon, P and Jones. S. 2009. The Economics of Smallholder Organic Contract Farming in Tropical Africa. *World Development,* 37(6), pp.1094–1104.

Brambilla, I and Porto, GG, 2011. Market structure, outgrower contracts, and farm output: Evidence from cotton reforms in Zambia. *Oxford Economic Papers*, 63(4), pp.740–766.

Briones, RM, 2015. Small farmers in high-values chains: Binding or relaxing constraints to inclusive growth? *World Development*, 72, pp.43–52.

Cahyadi ER and Waibel, H, 2011. *Do Smallholders Gain from Contract with an Oil Palm Company? Lessons Learned from Jambi, Indonesia.* Conference on International Research on Food Security, Natural Resource Management and Rural Development.

Cahyadi, ER and Waibel, H, 2013. Is contract farming in the Indonesian oil palm industry pro-poor? *Journal of Southeast Asian Economies*, 30(1), pp.62–76.

Cahyadi, ER and Waibel, H, 2016. Contract farming and vulnerability to poverty among oil palm smallholders in Indonesia. *Journal of Development Studies*, 52(5), pp.681–695.

Dihel, NC, Goswami, AG, Hollweg, CH and Slany, A, 2018. *How Does Participation in Value Chains Matter to African Farmers?* SSRN Scholarly Paper 3238346. Rochester, NY: Social Science Research Network.

Escobal, JA and Cavero. D, 2012. Transaction Costs, Institutional Arrangements and Inequality Outcomes: Potato Marketing by Small Producers in Rural Peru. *World Development,* 40, pp.329–41.

Freguin-Gresh, S, d’Haese, M, & Anseeuw, W, 2012. Demythifying contract farming: Evidence from rural South Africa. *Agrekon*, 51(3), pp.24–51.

Girma, J and Gardebroek, C, 2015. The Impact of Contracts on Organic Honey Producers' Incomes in Southwestern Ethiopia*. Forest Policy and Economics* 50, pp.259–68.

Herrmann, RT, 2017. Large-scale agricultural investments and smallholder welfare: A comparison of wage labor and outgrower channels in Tanzania. *World Development*, 90, pp.294–310.

Herrmann, R and Grote, U, 2015. Large-scale agro-industrial investments and rural poverty: Evidence from sugarcane in Malawi. *Journal of African Economies*, 24(5), pp.645–676.

Ito, J, Bao, Z and Su, Q, 2012. Distributional Effects of Agricultural Cooperatives in China: Exclusion of Smallholders and Potential Gains on Participation. *Food Policy,* 37(6), pp.700–09.

Jones, S and Gibbon. P, 2011. Developing Agricultural Markets in Sub-Saharan Africa: Organic Cocoa in Rural Uganda. *Journal of Development Studies,* 47(10), pp.1595–618.

Kumar, A, Roy, D, Trapathi, G, Joshi, PK and Adhikari, RP, 2016. *Can Contract Farming Increase Farmers’ Income and Enhance Adoption of Food Safety Practices?: Evidence from Remote Areas of Nepal*. IFPRI Discussion Paper 1524. Washington, D.C.: International Food Policy Research Institute (IFPRI).

Maertens, M and Swinnen, J, 2009. Trade, Standards, and Poverty: Evidence from Senegal. *World Development,* 37(1), pp.161–78.

Maertens, M, & Vande Velde, K, 2017. Contract-farming in staple food chains: The case of rice in Benin. *World Development*, 95, pp.73–87.

Michelson, HC, 2013. Small farmers, NGOs, and a Walmart world: Welfare effects of supermarkets operating in Nicaragua. *American Journal of Agricultural Economics*, 95(3), pp.628–649.

Mishra, AK, Kumar, A., Joshi, PK and D’souza, A, 2016. Impact of contracts in high yielding varieties seed production on profits and yield: The case of Nepal. *Food Policy*, 62, pp.110–121.

Miyata, S, Minot N and Hu, D, 2009. Impact of Contract Farming on Income: Linking Small Farmers, Packers, and Supermarkets in China. *World Development,* 37(11), pp.1781–90.

Munongo, S, 2012. Welfare Impact of Private Sector Interventions on Rural Livelihoods: The Case of Masvingo and Chiredzi Smallholder Farmers. *Russian Journal of Agricultural and Socio-Economic Sciences,* 10(10), pp.3–9.

Muriithi, BW, & Matz, J A, 2015. Welfare effects of vegetable commercialization: Evidence from smallholder producers in Kenya*. Food Policy*, 50, pp.80–91

Mwambi, MM, Oduol, J, Mshenga, P and Saidi, M, 2016. Does Contract Farming Improve Smallholder Income? The Case of Avocado Farmers in Kenya. *Journal of Agribusiness in Developing and Emerging Economies,* 6 (1), pp.2–20.

Narayanan, S, 2014. Profits from Participation in High Value Agriculture: Evidence of Heterogeneous Benefits in Contract Farming Schemes in Southern India. *Food Policy,* 44, pp.142–57.

Nhan, TQ, Gillette, R, Yutaka, T and Can ND, 2020. Impact of Outgrower Scheme on Yield,Output Price,and Income: A Rice-Farm-Level Study in the Mekong Delta,Vietnam. *Hitotsubashi Journal of Economics,* 61(1), pp.1–19.

Ramaswami, B, Birthal, PS and Joshi, PK, 2009. Grower Heterogeneity and the Gains from Contract Farming: The Case of Indian Poultry. *Indian Growth and Development Review,* 2(1), pp.56–74.

Rao, E and Qaim, M, 2011. Supermarkets, Farm Household Income, and Poverty: Insights from Kenya. *World Development,* 39(5), pp.784–96.

Saigenji, Y, 2012*. Contract Farming and Its Impact on Production Efficiency and Rural Household Income in the Vietnamese Tea Sector.* Faculty of Agricultural Sciences, Institute of Agricultural Economics and Social Sciences in the Tropics and Subtropics. University of Hohenheim, Germany.

Setboonsarng, S, Leung, P and Stefan, A, 2008. *Rice contract farming in Lao PDR: Moving from subsistence to commercial agriculture*. Tokyo: Asian Development Bank Institute.

Simmons, P, Winters, P and Patrick, I, 2005. An Analysis of Contract Farming in East Java, Bali, and Lombok, Indonesia. *Agricultural Economics,* 33(3), pp.513–25.

Sokchea, A and Culas RJ, 2015. Impact of Contract Farming with Farmer Organizations on Farmers’ Income: A Case Study of Reasmey Stung Sen Agricultural Development Cooperative in Cambodia. *Australasian Agribusiness Review,* 23.

Soullier, G and Moustier, P, 2018. Impacts of Contract Farming in Domestic Grain Chains on Farmer Income and Food Insecurity. Contrasted Evidence from Senegal. *Food Policy,* 79(August), pp.179–98.

Tefera, DA, and Bijman, J, 2021. Economics of Contracts in African Food Systems: Evidence from the Malt Barley Sector in Ethiopia. *Agricultural and Food Economics*, 9(1), 26.

Väth, SJ and Kirk, M. 2013. *Do Land Ownership and Contract Farming Matter? Evidence from a Large-Scale Investment in Ghana.* Fourth International Conference, September 22-25, 2013, Hammamet, Tunisia. African Association of Agricultural Economists (AAAE).

Wainaina, PW, Okello, JJ and Nzuma, JN, 2014. Blessing or Evil? Contract Farming, Smallholder Poultry Production and Household Welfare in Kenya, *Quarterly Journal of International Agriculture,* 53, pp.319–40.

Wang, H, Moustier, P and Loc, NTT, 2014. Economic Impact of Direct Marketing and Contracts: The Case of Safe Vegetable Chains in Northern Vietnam*. Food Policy*, 47, pp.13–23.

Warning, M and Key, N, 2002. The Social Performance and Distributional Consequences of Contract Farming: An Equilibrium Analysis of the Arachide De Bouche Program in Senegal. *World Development,* 30(2), pp.255–163.

Wendimu, MA, Henningsen, A and Gibbon, P, 2016. Sugarcane Outgrowers in Ethiopia: “Forced” to Remain Poor? *World Development,* 83, pp.84–97.

Winters, P, Simmons, P and Patrick, I, 2005. Evaluation of a Hybrid Seed Contract between Smallholders and a Multinational Company in East Java, Indonesia. *Journal of Development Studies,* 41, pp.62–89.

***Improved storage infrastructure (n=4)***

Aggarwal, S, Francis, E and Robinson, J, 2018. Grain Today, Gain Tomorrow: Evidence from a Storage Experiment with Savings Clubs in Kenya. *Journal of Development Economics,* 134(September), pp.1–15.

Casaburi, L, Glennerster, R, Suri, T and Kamara, S, 2017. Providing Collateral and Improving Product Market Access for Smallholder Farmers: A Randomized Evaluation of Inventory Credit in Sierra Leone. *Harvard Dataverse.*

Kizito, AM and Kato, E, 2018. Does linking farmers to markets work? Evidence from the World Food Programme’s Purchase for Progress satellite collection points initiative in Uganda. *African Journal of Agricultural and Resource Economics,* 13(2), pp.169–181.

Lentz, E and Upton, J, 2016. Benefits to smallholders? Evaluating the World Food Programme's Purchase for Progress pilot. *Global Food Security,* 11, pp.54–63.

*Note: Studies are mapped approximately to the intervention type and some studies may consist of components relevant to more than one intervention type. These nuances will be documented in our final review.*

# **Appendix 7. Provisional data extraction form**

| **Variable group** | **Variable** | **Description** |
| --- | --- | --- |
| Publication Information | Study ID | The unique ID code that is assigned to each included study |
|  | Estimate ID | The unique ID code that assigned to each individual estimate |
|  | Study status | Select one of the following:  i) Completed; ii) Protocol; iii) Ongoing |
|  | Author Name | Authors last names [Open Answer] |
|  | Year of Publication | Year published (publication date, not preprint or first online publication dates) |

| Intervention Information | Intervention code | Choose one or more intervention code(s) for each corresponding effect size:  i) Farm to market transport; ii) Output market info; iii) New marketplaces & alternative marketing opportunities; iv) Contract farming; v) Improved storage infrastructure |
| --- | --- | --- |
|  | Intervention sub-group | Choose one or more intervention sub-group code(s) for each corresponding effect size:  ● Farm to market transport infrastructure  Domestic transport infrastructure  Export infrastructure  ● Output market info  Direct transmission or provision of output market info (mobile and internet)  Direct transmission or provision of output market information  (Other forms of communication, e.g., radio)  Investments in information and communication technologies infrastructure  ● New marketplaces & alternative marketing opportunities  Online commodity exchanges and mobile-based marketplaces  Alternative physical marketplaces  Arranged or committed offers from buyers  ● Contract farming  Fixed-price and price guarantee contracts  Production-management contracts  Input or credit-supply contracts  Other contract types  ● Improved storage infrastructure & technologies  Improved storage technologies for transit                 Improved on-farm storage infrastructure and technologies  Storage deposit systems |
|  | Country | Country of intervention |
|  | Exposure to intervention (in months) | For how long are the observations exposed to the intervention? |
|  | Evaluation period (in months) | The total number of months elapsed between the end of an intervention and the point at which an outcome measure is taken post intervention, or as a follow-up measurement.  If less than one month, use decimals (e.g., measurement immediately after the intervention end would be coded as 0, one week would be .25, etc.) |
|  | Intervention description | Provide detailed description of the intervention and its different components such that a reader could easily understand what happened. Include page numbers for quick reference. If two or more interventions are being evaluated, please provide descriptions for each intervention arm under separate rows. |
|  | Type of agricultural product | What is the agricultural product that the intervention aims to increase market access to. Provide a short description (e.g. Livestock-Unspecified, Beef/cattle, Cassava, Cocoa, Coffee, Cotton, etc.) |
|  | Cost | Report any cost data provided or comments on cost effectiveness, include the authors' comments on cost data even if quantifications are not provided. Provide details of what the cost relates to or how they have been calculated if possible. Include any information identified from cited documents or linked studies. |

| Method information | | Evaluation Design | Select one of the options below:  1. Experimental (defined as prospective randomised assignment, where randomisation is implemented by researchers (or by decision makers in the context of an evaluation study)  2. Quasi-experimental (including natural experiments and non-randomised studies). | |  |
| --- | --- | --- | --- | --- | --- |
|  |  | Evaluation Method | If Experimental, then select:  Randomised controlled trial  If Quasi-experiment or natural experiment, then select:  Natural experiment in which exposure to treatment is random  Regression Discontinuity Design (RDD)  Difference-in-Differences (DID) / Fixed effects estimation  Instrumental variable (IV) estimation  Endogenous treatment-effects models (including endogenous switching regression, and other methods synonymous to the Heckman two step model)  Statistical matching (includes PSM or statistical weighting)  Interrupted time series (ITS)  Synthetic controls | |  |
|  |  | Large scale/spillover | Is the analysis a “large scale experiment” or capturing spill over effects? If yes, describe the approach used. [Open Answer] | |  |
|  |  | GE | Does the study present general equilibrium estimates or control for spillovers by design? If yes, describe the approach [Open Answer] | |  |
|  |  | Additional Methods | Select additional method if any. If none, select not applicable. [Open Answer] | |  |
| Estimate  Information | Analysis type for this effect size | | | Free text, what type of analysis was used (Regression, 2SLS, ANCOVA, etc.) | |
|  | Estimate Type | | | Type of data for this effect size: 1 = Continuous - means and SDs, 2 = Continuous - mean difference and SD, 3 = Dichotomous outcome - proportions, 4 = Regression data - dichotomous outcome, 5 = Regression data - continuous outcome | |
|  | Treatment Effect | | | 1=Intention to Treat (ITT), 2=Average Treatment Effect on the Treated (ATET), 3=Average Treatment Effect (ATE) 4 = Local Average Treatment Effect (LATE) | |
|  | Unit of analysis | | | What is the unit of analysis? UOA for this effect size: 1= Individual, 2= Household, 3= Group (e.g., community organisation), 4= Village, 5 = Other, 6 = Not clear | |
|  | Source | | | Note the page number, table number, column, and row you used to extract the data  [Open Answer] | |
| Treatment variable information | Treatment | | | Record the treatment variable as written in the model (e.g., the variable name the author uses, such as ("Intervention x Time")  [Open Answer] | |
|  | Treatment type | | | Describe the types of treatment variable used: i) binary; ii) continuous; iii) categorical; iv) other | |
|  | Comparison | | | 1=No intervention (service delivery as usual), 2=Other intervention, 3=Pipeline (waitlist) control (still service delivery as usual) | |
|  | Describe Comparison Group | | | Describe the comparison group [Open Answer] | |
|  | Subgroup | | | Is this analysis of a subgroup or estimating heterogeneous effects?  0=no, 1=yes | |
|  | Subgroup information | | | Describe the subgroup or variable interacted with the treatment variable if applicable (e.g., boys, girls).  If no subgroup, select not applicable [Open Answer] | |

| Outcome  Information | Outcome description | Record the outcome for the corresponding effect size. Use this open answer field to enter, in the author’s own words, a description of the outcome. Be selective and concise with the excerpts being transcribed here as to ensure accurate and precise descriptions of the outcome. To the extent possible, be sure to include numbers, units, population, and comparators. Include page numbers with every excerpt extracted. |
| --- | --- | --- |
|  | Outcome codes | Choose an outcome code for each corresponding effect size: i) Immediate; ii) Agricultural production; iii) Intermediate; iv) Off-farm outcomes (Agriculture only); v) Non/off-farm outcomes; vi) Welfare |
|  | Outcome sub-group | Choose an outcome sub-group code for each corresponding effect size:  ● Immediate           Transaction costs           Aggregation           Use/ adoption of improved inputs, technologies & practices           Farm investment           Access to credit           Prices  ● Agricultural Production            Yields            Volume            Quality            Produce type  ● Intermediate            Sales            Farm income  ● Off-farm outcomes (Agriculture only)            Off-farm income            Labour market outcomes  ● Non/off-farm outcomes            Non/off-farm income            Labour market outcomes  ● Welfare           Total household income and wealth           Food and nutrition security |
|  | Outcome description | Record the outcome for the corresponding effect size. Use this open answer field to enter, in the author’s own words, a description of the outcome. Be selective and concise with the excerpts being transcribed here as to ensure accurate and precise descriptions of the outcome. To the extent possible, be sure to include numbers, units, population, and comparators. Include page numbers with every excerpt extracted. |
|  | Post-intervention or change from baseline? | 0 = Post-intervention, 1 = Change from baseline |

| Estimate data | Mean treatment | Outcome mean for the treatment group |
| --- | --- | --- |
|  | SD treatment | Outcome standard deviation for treatment group |
|  | Mean Control | Outcome mean for the comparison group |
|  | SD Control | Outcome standard deviation for control group |
|  | Mean difference | Overall mean difference (treatment - control) |
|  | SE difference | Standard error of the overall mean difference |
|  | Tstat difference | t-statistic of mean difference |
|  | p-value difference | p-value of mean difference |
|  | Odds ratio | Odds ratio reported in the study |
|  | SE odds ratio | Odds ratio standard error reported in the study |
|  | Risk ratio | Risk ratio reported in study |
|  | SE risk ratio | Risk ratio standard error |
|  | Coeff reg | Report the regression coefficient of the treatment effect |
|  | SE reg | Report the associated standard error of the regression coefficient. |
|  | Tstat reg | Report the associated t statistic of the effect size (coefficient/SE) |
|  | CI_LB reg | Report the associated Lower bound of the 95% Confidence interval of the effect size. If CI is reported for a different confidence level, indicate that in the notes section. |
|  | CI_UP reg | Report the associated Upper bound of the 95% Confidence interval of the effect size. If CI is reported for a different confidence level, indicate that in the notes section. |
|  | P value exact | Exact p value if given, if not, record as written in the manuscript (e.g., p < .001, or p > .05) |
|  | Clusters treatment | Number of clusters - treatment group |
|  | Clusters control | Number of clusters -  control group |
|  | Clusters total | Number of clusters - total sample |
|  | N treatment | Sample size - treatment group |
|  | N control | Sample size - control group |
|  | N total | Sample size - total sample |
|  | periods (1 if cross sectional) | Record how many time-period there are in the evaluation (e.g., cross section is 1, panel data with 3 measurements is 3) |
|  | Does the sample size need to be corrected? | Often in panel data, models will report number of observations rather than number of participants. In this column you will indicate 1="Yes" if the sample size needs to be divided by the number of periods, and 0="No" if either it is cross-sectional data, or if the authors have already divided the number of observations by the number of panel assessments and thus no correction is necessary. |

# **Appendix 8. Calculating standardised effects**

***Continuous outcomes***

For studies reporting regression results for continuous outcomes, we will standardise the effect sizes following the approach suggested by Keef and Roberts (2004). This includes dividing the regression coefficient (β) by the pooled standard deviation (SD) of the outcome.

$SMD= \frac{\beta}{{SD}_{pooled}}$ (i)

When using parsimonious regression specifications, this approach is analogous to Cohen’s d (d), which is the difference in means between the treatment and control (or comparison) group divided by the pooled SD of the outcome (i.e. the standardised mean difference). Because Cohen's *d* can be biased in cases where sample sizes are small, in all cases we will simply adjust *d* using Hedges' method. This transformation adjusts Cohen's *d* to Hedges' *g* using the following formula (Ellis, 2010):

$g\cong d(1-\frac{3}{4\left( n_{T}+n_{C} \right)- 9})$ (ii)

Where *n* denotes the sample size of the treatment (n_T_) and control (n_C_) groups. If the intervention is expected to change the *SD* of the outcome variable, we will use the *SD* of the control group to compute d instead. If the study does not report the pooled *SD* but information about sample size is available for both the treatment and control groups, we will use regression coefficients and standard errors (*SE*s) or *t* statistic (t) to calculate the following:

$d=t\sqrt{\frac{1}{n_{T}}+\frac{1}{n_{C}}}$ (iii)

Alternatively, when only information on the total sample size (*N*) is available, we will use the following formula suggested by Polanin et al. (2016):

$d= \frac{2t}{\sqrt{N}}$ (iv)

${Var}_{d}=\frac{4}{N}+ \frac{d^{2}}{4N}$   (v)

Here we will calculate the *t*-statistic (*t*) by dividing the coefficient by the *SE*. If the authors only report confidence intervals (CI) and no *SE*, we will calculate the *SE* from the confidence intervals:

$SD=\sqrt{N} \frac{(upper CI-lower CI)}{I}$ (vi)

where I is 3.29 for estimates using 90% confidence intervals, 3.92 for 95% confidence intervals, and 5.15 for 99% confidence intervals.

In cases in which significance levels are reported rather than *t* or a beta coefficient (b) with the associated SE, then we will impute *t* using a t-distribution table. If the precise probability value is not reported, then we will assume the following:

*Prob > 0.1: t = 0.5*

*0.1 ≥ Prob > 0.05: t = 1.645*

*0.05 ≥ Prob > 0.01: t = 1.960*

*0.01 ≥ Prob:  > 0.001: t = 2.576*

*Prob: ≥   0.001: t = 3.291*

In some cases, the studies we include in the review may not report a regression coefficient, but the group means ($\bar{X}$) and pooled SD for treatment and control group at follow up only (*p* + 1). Here we will calculate d using formulae provided in Borenstein et al. (2009):

$d= \frac{\bar{X}_{T_{p+1}}- \bar{X}_{C_{p+1}}}{{SD}_{p+1}}$ (vii)

If the study does not report the pooled SD, it is possible to calculate it using the following formula:

${SD}_{p+1}= \sqrt{\frac{\left( n_{T_{p+1}}-1 \right){SD}_{T_{p+1}}^{2}+ (n_{C_{p+1}}-1){SD}_{C_{p+1}}^{2}}{n_{T_{p+1}}n_{C_{p+1}}-2}}$  (viii)

For studies reporting the difference in treatment and control group means and the pooled SDs at baseline (*p*) and follow up (*p* + 1):

  $d= \frac{\Delta\bar{X}_{T_{p+1}}- \Delta\bar{X}_{T_{p}}}{{SD}_{p}}$ (ix)

Finally, for studies reporting mean differences between treatment and control group, standard error (SE) and sample size (n):

   $d=\frac{\Delta\bar{X}_{T_{p+1}}}{SE\sqrt{n}}$ (x)

***Outcomes measured as proportion of individuals***

If outcomes are reported in proportions of individuals, we will calculate the Cox-transformed log odds ratio effect size (Sánchez-Meca et al., 2003):

$d=LogOddsRatio \frac{\sqrt{3}}{\pi}$ (xi)

***Outcomes measured as proportion of events or days***

If outcomes are reported based on proportions of events or days, we will use the standardised proportion difference effect size:

$d= \frac{w_{T} - w_{C}}{SD(w)}$ (xii)

Where $w_{T}$ is the proportion in the treatment group and $w_{C}$ the proportion in the comparison group, and the denominator is given by:

$SD\left( w \right)=\sqrt{w1-w}$ (xiii)

Here w is the weighted average of $w_{T}$ and $w_{C}$:

$w= \frac{n_{T}w_{T} + n_{C}w_{C}}{n_{T}+n_{C}}$ (xiv)

# **Appendix 9. Criteria determining selection of effect estimates for data extraction**

We will extract effects reported across different interventions, outcomes and subgroups within a study. We will address dependent effect sizes using data processing and selection techniques. We will utilise several criteria to select one effect estimate per outcome per study:

- Where studies report effects from multiple model specifications, we will use the author's preferred model specification. Only if the preferred specification is unclear, we will use the most efficient estimate of the treatment effect (measured by the one with the largest t-value). This reflects regression adjustments in designs, such as RCTs, IV, RDDs, are usually made on grounds of model efficiency but explorative specifications (e.g. including interaction terms, higher order terms, etc.) may not be the most efficient estimates (in fact they could be highly inefficient) where the added terms are not significant.
- Where studies report effects from multiple estimators, we will use the author's preferred specification. Only if the preferred specification is unclear, we will use the specification that appears most robust to falsification tests (e.g. according to sensitivity analysis for propensity score matching or placebo tests for difference-in-difference estimators).
- Where different studies report on the same programme but use different samples (e.g., from different regions), we will include both estimates, treating them as independent samples, provided effect sizes are measured relative to separate control or comparison groups.
- Where studies report evidence according to subgroups of participants, we will record and report data on relevant subgroups separately.
- For studies with outcome measures at different time points, we will synthesise short- and long-term outcomes separately, following Rue and colleagues (2013).
- When studies include multiple outcome measures to assess related outcome constructs, we will follow our pre-specified preferred outcome order (described below) without reference to the results.
- Where studies report multiple outcome subgroups for the same outcome construct, but do not present an effect for the full sample, we may calculate a “synthetic effect size” using the sample-weighted average, and applying appropriate formulae to recalculate variances (Borenstein and et al., [2009](https://onlinelibrary.wiley.com/doi/10.1002/cl2.1180#cl21180-bib-0013), ch. 24).
- If studies include multiple treatment arms with only one control group and the treatments represent separate treatment constructs, we will calculate the effect size for treatment A versus control and treatment B versus control and include them in separate meta-analyses according to the intervention type.

This also includes criteria prioritising specific outcome measures within included studies:

- For outcomes related to transaction costs, inputs, technologies or practices, and farm investment, our analysis will prioritise synthesising outcomes using composite or aggregate indicators. If a study does not report a composite measure, we will use the outcome that most closely relates to the intervention type (e.g. transport costs for farm to market interventions) or perform outcome mapping to identify the outcome in each study that appears most frequently across studies.
- Where an intervention targets or concerns one specific commodity, our analysis will use commodity specific outcomes for price, production, and intermediate outcomes. If the intervention targets and reports outcomes for more than one commodity, we will use the most frequent type of commodity specific outcome reported across studies. If a study reports outcomes for both arable and permanent crops, we will select one prioritised arable crop and one prioritised permanent crop to perform sensitivity analysis on the type of crop prioritised for this sample of studies. We will also distinguish between staple and cash crops, again taking the most frequent staple or cash crop reported across studies as the prioritised outcome.
- Specific preferences for measures of outcome constructs are also outlined in the table below. We will consider, where possible, creating combined and separate meta-analysis for different measures of the same outcome construct or control for groups of outcome measures in a meta-regression.

The number of possible ways any outcome construct can be measured is often large. The list of priority outcomes in the table below are unlikely to be entirely comprehensive given it only accounts for some common measures which are presented simultaneously in studies (which creates the need to choose a preferred outcome for the synthesis). We will consult subject experts, without reference to the results of a study, to establish an order of preference should studies present multiple measurements of an outcome construct that is not already captured by the priority criteria established in this protocol.

| **Outcome Type** | **Outcome** | **Description** |
| --- | --- | --- |
| Immediate outcomes | Transaction costs | In order of priority, we will extract data for any measures of aggregate costs, transport costs, search and information costs, bargaining costs, monitoring and enforcement costs: i) for outputs ii) for inputs. |
|  | Aggregation | In order of priority, we will extract data for any measures of i) incidence of membership of farmer organisations ii) number of farmer organisations. |
|  | Use/ adoption of improved inputs, technologies & practices | In order of priority, we will extract data for outcomes that measure i) volume of inputs per unit of land area; ii) measures of the number or volume of inputs, technologies or practices used (e.g. average number of sprays per season); iii) binary measures indicating the prevalence of input etc. use. |
|  | Farm investment | In order of priority, we will extract data for outcomes that measure i) the expenditure on inputs/technology, ii) willingness to invest in inputs/technology |
|  | Access to credit | In order of priority, we will extract data for outcomes that measure i) the total amount borrowed; ii) whether farmers indicate they have borrowed money for farm investment in a preceding period of time. |
|  | Prices | In order of priority, we will extract data for outcomes that measure i) average price sold; ii) average price expected/committed. |
|  | Crop losses | In order of priority, we will extract data for outcomes that measure i) the weight of crop losses per hectare; ii) the total weight of crop losses. We will prioritise objective measures over self-reported measures of post-harvest losses. |
| Agricultural Production outcomes | Yields | In order of priority, we will extract data for outcomes that measure i) output per land unit; ii) output per worker. |
|  | Volume | In order of priority, we will extract data for outcomes that measure i) the total weight of production; ii) share or amount of land cultivated iii) the number of farmers cultivating. |
|  | Quality | In order of priority, we will extract data for outcomes that measure i) laboratory verified testing ii) visual inspections |
|  | Produce type | In order of priority, we will extract data for outcomes that measure i) the proportion of land cultivated; ii) the amount of land cultivated; iii) the total weight of production; iv) the number of farmers cultivating or growing a crop; v) binary measures of the incidence crop cultivation. |
| Intermediate outcomes | Sales | In order of priority, we will extract data for outcomes that measure i) the proportion of total cultivated produce sold; ii) the volume of produce sold; iii) the total number of sales; iv) indicator of whether a farmer sells some of their produce, or not. |
|  | Farm income | In order of priority, we will extract data for outcomes that measure i) net farm income; ii) revenue or monetary value of produce sold. We will extract data for gross farm income where net income is not reported. |
| Off-farm outcomes (Agriculture only) | Off-farm income | In order of priority, we will extract data for outcomes that measure net off-farm income (from activities that occur beyond the farm owned by the household). We will extract data for gross farm income where net income is not reported. |
|  | Labour market outcomes | In order of priority we, we will extract data for i) hours of off-farm work (from activities that occur beyond the farm owned by the household); ii) binary indicators of employment or work status.  We will extract data for corresponding off-farm outcomes separately too. |
| Non/off-farm outcomes | Non/off-farm income | In order of priority, we will extract data for outcomes that measure net income from non-farm activities. We will extract data for corresponding off-farm income outcomes separately too. We will extract data for gross income where net income is not reported. |
|  | Labour market outcomes | In order of priority we, we will extract data for i) hours of non-farm work; ii) binary indicators of employment or work status.  We will extract data for corresponding off-farm outcomes separately too. |
| Welfare outcomes | Total income and wealth | In order of priority, we will extract data for outcomes that measure i) total household income; ii) total household expenditure or consumption; iii) asset or wealth index. |
|  | Food and nutrition security | In order of priority, we will extract data for outcomes that measure i) food and nutrition security indices and composite scores of the extent to which households have food to meet basic dietary needs; ii) measures of food consumption and nutritional intake, such as caloric intake, meal frequency, minimum meal intake/acceptable diet, consumption of iron-rich or iron-fortified foods, vitamin supplementation, fruit and vegetable intake, micronutrient intake; iii) reports of insufficient food. We will extract data for food and nutrition security outcomes separately. |

# **Appendix 10. Risk of Bias Assessment Tool**

## ***Tool for Randomised Experiments***

|  | **Coder** | **General** | **General** | **General** | **General** |
| --- | --- | --- | --- | --- | --- |
| **Question** |  | ID | Study first author | Time taken to complete assessment | Design type: What type of study design is used? |
| **Coding format** |  | EPPI ID | Open answer | Minutes | 1= Randomised controlled trial (RCT) (random assignment to households/individuals) or quasi-RCT  2= Cluster-RCT (quasi-RCT) |
| **Criteria** |  |  |  |  | - |
| **Decision rule** |  |  |  |  |  |
| Response | Core Team |  |  |  |  |
| **Response** | **Core Team** |  |  |  |  |
|  |  |  |  |  |  |
| **General** | **General** | **General** | **General** | **General** | **General** |
| Methods used for analysis: Which methods are used to control for selection bias and confounding? | Design and analysis method description | Study population | Type of comparison group | Type of comparison group (if other) | Ethical clearance |
| 1 = Statistical matching (PSM, CEM, covariate matching) 2 = Difference in differences (DID) estimation methods 3 = IV-regression (2-stage least squares or bivariate probit) 4 = Heckman selection model 5 = Fixed effects regression 6 = Covariate adjusted estimation 7 = Propensity weighted regression 8 = Comparison of means 9 = Other (please state) | Open answer | Open answer | 1=No intervention (service delivery as usual)  2=Other intervention  3=Pipeline (wait-list) control (still service delivery as usual) | Open answer | Open answer |
| - | Briefly describe the study design and analysis method undertaken by the authors. | Provide any details in the paper that describe how the study population was selected, covering:  a) How is the population selected? what is the sampling strategy to recruit participants from that population into the study? b) What are the characteristics of that study participants? c) Was this a pilot program aimed at being scaled up? d) Were there specific factors of success or failure in the implementation? | Indicate type of comparison group |  | Provide any details of ethical research clearances granted. Report unclear if this information is not available. |

|  |  |  |  |  |  |
| --- | --- | --- | --- | --- | --- |
| **General** | **1: Assignment mechanism - Assessment** | **1: Assignment mechanism - Justification** | **2: Unit of analysis - Assessment** | **2: Unit of analysis - Justification** | **3: Selection bias - Assessment** |
| Study registration | Assignment mechanism: Was the allocation or identification mechanism random or as good as random? | Assignment justification | Unit of analysis: Is unit of analysis in cluster allocation addressed in standard error calculation ? | Method used to address differences between UoA and unit of data collection | Selection bias Was any differential selection into or out of the study (attrition bias) adequately resolved? |
| Open answer | 1= Yes, 2 = Probably Yes, 3 = Probably No, 4 = No, 8 = Unclear | Open answer | 1=Yes 2=No 3=Not reported/unclear 4=Not applicable | Open answer | 1= Yes, 2 = Probably Yes, 3 = Probably No, 4 = No, 8 = Unclear |
| Provide any detials of study registration, inlcuding registry IDs, etc. | a) The authors describe a random component in sequence generation/ randomization method (e.g. lottery, coin toss, random number generator) and assignment is performed for all units at the start of the study centrally or using a method concealed from participants and intervention delivery   b) If public lottery is used for the sequence generation, authors provide detail on the exact settings and participants attending the lottery.  c) If a special randomization procedure is used to ensure balance, it is well described and justified given the study setting (stratification, pairwise matching, unique random draw, multiple random draws etc).   d) A balance table is reported suggesting that allocation was random between all groups including subgroup receiving different treatment within control or treatment groups (if the comparison is relevant for this assessment). | Justification for coding decision  (Include a brief summary of justification for rating, mentioning your response to all sub questions, cite relevant pages). | Score "Yes" if Unit of analysis (UoA) = Unit of randomisation (UoR) **OR** if UoA ≠ UoR and standard errors are clustered at the UoR level OR data is collapsed to the UoR level   Score "Not reported/unclear" if not enough information is provided on the way the standard errors were calculated or what the unit of analysis is.   Score "Not applicable" if it is not a cluster RCT.   Score "No" otherwise. |  | Score "Yes" if there is no attrition or attrition falls into the green zone (see figure A.1 below) and the study establishes that attrition is randomly distributed (e.g. by presenting balance by key characteristics across groups) **AND** if survey respondents were randomly sampled.    Score "Probably yes" if attrition falls into the green zone **AND** if survey respondents were randomly sampled.    Score "Unclear" if there is an attrition problem but no information provided on the relationship between attrition and treatment status, OR if there is not enough information on how the population surveyed was sampled.    Score "Probably no" if there is attrition which is likely to be related to the intervention OR there is some indication that the survey respondents were purposely sampled in a way that might have led the sampling to be different between treatment and control groups, or attrition falls into the yellow zone.    Score "No" if attrition falls into the red zone. |
|  | Score “Yes” if all criterion a), b), c) and d) are satisfied.    Score "Probably Yes" if only criterion a) and b) are not satisfied OR if only criteria c) is not satisfied.    Score “Unclear” if d) is not satisfied because no balance table is reported.    Score "Probably No" if d) is not satisfied because there is no balance table reported and there is evidence suggesting a problem in the randomization, such as baseline coefficients in a diff-in-diff regression table are very different or sample size is too small for the procedure used (using stratification when there are less than two units for each intervention and control group in each strata can lead to imbalance).     Score “No” if d) is not satisfied because there are large imbalances concerning a large number of variables, providing evidence that the assignment was not random. If this is scored as no, use the NRS tool. |  |  |  |  |

|  |  |  |  |  |  |
| --- | --- | --- | --- | --- | --- |
| **3: Selection bias - Justification** | **3: Confounding - Assessment** | **3: Confounding - Justification** | **4: Deviations from intended interventions - Assessment** | **4: Deviations from intended interventions - Justification** | **5. Performance bias - Assessment** |
| Selection bias justification | Confounding and group equivalence: Was the method of analysis executed adequately to ensure comparability of groups throughout the study and prevent confounding | Confounding justification | Deviations from intended interventions: Spill-overs, cross-overs and contamination: was the study adequately protected against spill-overs, cross-overs and contamination? | Deviations justification | Performance bias: Was the process of monitoring individuals unlikely to introduce motivation bias among participants? |
| Open answer | 1= Yes, 2 = Probably Yes, 3 = Probably No, 4 = No, 8 = Unclear | Open answer | 1= Yes, 2 = Probably Yes, 3 = Probably No, 4 = No, 8 = Unclear | Open answer | 1= Yes, 2 = Probably Yes, 3 = Probably No, 4 = No, 8 = Unclear |
| Justification for coding decision  (Include a brief summary of justification for rating, mentioning your response to all sub questions, cite relevant pages). | a) Baseline characteristics are similar in magnitude;  b) Unbalanced covariates at the individual and cluster level are controlled in adjusted analysis;  c) Adjustments to the randomization were taken into account in the analysis (stratum fixed effects, pairwise matching variables)? (Bruhn and McKenzie 2009) | Justification for coding decision  (Include a brief summary of justification for rating, mentioning your response to all sub questions, cite relevant pages). | a) There was no implementation issues that might have led the control participants to receive the treatment (implementer's mistake).  b) The intervention is unlikely to spill-over to comparisons (e.g. participants and non-participants are geographically and/or socially separated from one another and general equilibrium effects are not likely) or the potential effects of spill overs were measured (e.g. variation in the % of unit within a cluster receiving the treatment).  c) There is no risk of contamination by external programs: the treatment and comparisons are isolated from other interventions which might explain changes in outcomes.   d) There is nothing in the surveys that might have given the control participants an idea of what the other group might receive OR they did but there is no risk that this has changed their behaviours; AND the survey process did not reveal information to the control group that they did not have before (e.g. the study aims to measure increase in take up of a service or product that participants might not know about)  Authors might put something in place in the design of the study that allows to control for that survey effect (e.g. a pure control with no monitoring except baseline end line) | Justification for coding decision  (Include a brief summary of justification for rating, mentioning your response to all sub questions, cite relevant pages).    For example, intervention groups are geographically separated, authors use intention to treat estimation or instrumental variables to account for non-adherence, and survey questions are not likely to expose individuals in the control group to information about desirable behaviours (‘survey effects’). | a) The authors state explicitly that the process of monitoring the intervention and outcome measurement is blinded and conducted in the same frequency for treatment and control groups, or argue convincingly why it is not likely that being monitored could affect the performance of participants in treatment and comparison groups in different ways (such as resulting in Hawthorne or John Henry effects).    b) The outcome is based on data collected in the context of a survey, and not associated with a particular intervention trial, or data are collected from administrative records or in the context of a retrospective (ex post) evaluation. |
|  | Score “Yes” if criterion a) and b) are satisfied;    Score "Probably yes" if a) is not satisfied but b) is satisfied and imbalances are small in magnitude OR if only a) is satisfied.     Score “Unclear” if no balance table is provided or if imbalances are controlled for but they are very large in magnitude and assignment mechanism is not coded as "Yes" or "Probably yes"    Score "Probably no" if a) and b) are not satisfied and the magnitude of imbalances are small    Score “No” if a) and b) are not satisfied and the magnitude of imbalances are large and covariates are clear determinant of the outcomes. |  | Score “Yes” if criterion a), b), c) and d) are satisfied;    Score "Probably yes" if there is no obvious problem but there is no information reported on potential risks related to spill overs, contamination, or survey effects in the control group OR if there were issues with spill-overs but they were controlled for or measured.    Score “Unclear” if spill-overs, cross-overs, survey effects and/or contamination are not addressed clearly.    Score "Probably no" if any of the criterion a), b), c) or d) are not satisfied but the scale of the issue is not clear.    Score “No” if any of the criterion a), b), c) or d) are not satisfied and happened at a large scale in the study. |  | Score “Yes” if either criterion a) or b) are satisfied;    Score "Probably yes" if the study is based on data collected during a trial and there is no obvious issue with the monitoring processes but authors do not mention potential risks.    Score “Unclear” if it is not clear whether the authors use an appropriate method to prevent Hawthorne and John Henry Effects (e.g. blinding of outcomes and, or enumerators, other methods to ensure consistent monitoring across groups). Hawthorne effects may result where participants know that they are being observed and John Henry Effects may result from participant knowledge of being compared.    Score "Probably no" if there was imbalance in the frequency of monitoring in intervention groups, which might have influenced participants' behaviours.    Score "No" if neither criterion a) or b) are satisfied. |

|  |  |  |  |  |  |
| --- | --- | --- | --- | --- | --- |
| **5. Performance bias - Justification** | **6. Outcome measurement bias - Assessment** | **6. Outcome measurement bias - Justification** | **7. Reporting bias - Assessment** | **8. Reporting bias - Justification** | **9. Other bias - Assessment** |
| Performance bias justification | Outcome measurement bias: Was the study free from biases in outcome measurement? | Outcome measurement justification | Analysis reporting: Was the study free from selective analysis reporting? | Analysis reporting justification | Is the study free from other sources of bias? |
| Open answer | 1= Yes, 2 = Probably Yes, 3 = Probably No, 4 = No, 8 = Unclear | Open answer | 1= Yes, 2 = Probably Yes, 3 = Probably No, 4 = No, 8 = Unclear | Open answer | 1= Yes, 4 = No |
| Justification for coding decision  (Include a brief summary of justification for rating, mentioning your response to all sub questions, cite relevant pages). | a) Outcome assessors are blinded or the outcome measures are not likely to be biased by their judgement.   b) For self-reported outcomes: respondents in the intervention group are not more likely to have accurate answers due to recall bias;  c) For self-reported outcomes: respondents do not have incentives to over/under report something related to their performance or actions, OR researchers put in place mechanisms to reduce the risk of reporting bias (researchers not strongly involved in the implementation of the program and it is clear that their answers to the survey will not affect what they receive in the future) OR authors have measured the risks of bias through falsification tests or measuring the effect on placebo outcomes in cases where there was a risk of reporting bias.  d) Timing issue: the data collection period did not differ between intervention and comparison group, the baseline data is not likely to be affected by the beginning of the intervention or affects a small percentage of the study participants. | Justification for coding decision  (Include a brief summary of justification for rating, mentioning your response to all sub questions, cite relevant pages). | a) A pre-analysis plan or trial protocol is published and referred to or the trial was pre-registered or the outcomes were pre-registered;  b) Authors report results corresponding to the outcomes announced in the methods section (there is no outcome reporting bias);  c) Authors report results of unadjusted analysis and intention to treat (ITT) estimation, alongside any adjusted and treatment-on-the-treated/complier-average-causal-effects analysis.)  d) Authors use the appropriate analysis method (use baseline data when available) and different treatment arms are differentiated in the analysis  e) Authors have reported all the analysis which could help understand the results and no other bias is assessed as unclear due to the lack of an important analysis (e.g. a balance table or a subgroup analysis) | Justification for coding decision  (Include a brief summary of justification for rating, mentioning your response to all sub questions, cite relevant pages). |  |
|  | Score “Yes” if criterion a), b), c) and d) are satisfied:    Score "Probably yes" if there is a small risk related to any of a), b), c) or d) and there is no more information provided to to justify the absence of bias OR if there was a high risk of bias but authors have either controlled it in their design or measured it with a placebo outcomes.    Score “Unclear” if it there is a high risk related to any of a), b), c) or d) and there is no more information provided to to justify the absence of bias.     Score "Probably no" if there are high risk related to a), b), c) or d) and it is clear that authors were not able to control for this bias.    Score “No” if there is evidence of bias. |  | Score "Yes" if all the criterion a), b), c), d), and e) are satisfied;  Score "Probably yes" if all the conditions are met except a), or if all the conditions are met but there is some element missing that could have helped understand the results better (e);  Score "Unclear" if there is not enough information to determine that there is an analysis missing;  Score "Probably no" if any of the criterion b), c) or d) are not satisfied;  Score "No" if any of the criterion b), c) or d) are not satisfied and there is evidence that the analysis results would be different because large imbalances were not controlled for, compliance was very low and ITT estimation was not reported or different treatment arms were pooled. |  |  |

|  |  |  |  |  |  |
| --- | --- | --- | --- | --- | --- |
| **9. Other bias - Justification** | **10. Blinding - observers - Assessment** | **10. Blinding - observers - Assessment** | **10. Blinding - analysts - Assessment** | **10. Blinding - method(s)** | **11. External validity - Assessment** |
| Other bias justification | Blinding of participants? | Blinding of outcome assessors? | Blinding of data analysts? | Method(s) used to blind | External validity |
| Open answer | 1=Yes 2=No 8=unclear 9= N/A | 1=Yes 2=No 8=unclear 9= N/A | 1=Yes 2=No 8=unclear 9= N/A | Open answer (including describe method of placebo control)No 9= N/A | Open answer |
| Justification for coding decision  (Include a brief summary of justification for rating, mentioning your response to all sub questions, cite relevant pages). For example, information is collected using a different survey instrument in different intervention groups; measurement of the intervention received is unclear. | If there is no information, code NO. If there is information but it is ambiguous, code UNCLEAR. | If there is no information, code NO. If there is information but it is ambiguous, code UNCLEAR. | If there is no information, code NO. If there is information but it is ambiguous, code UNCLEAR. | Describe method(s) used to blind | a) What do authors say about external validity? |
|  |  |  |  |  | Include all information that can help assess the external validity of the results. |

## ***Tool for Quasi-Experimental Designs***

| **Code** | **Coder** | **General** | **General** | **General** |
| --- | --- | --- | --- | --- |
| **Question** |  | Time taken to complete assessment | Study first author | Outcome |
| **Coding** |  | Minutes | Open answer | Open answer |
| **Criteria** |  |  |  |  |
| **Decision-rules** |  |  |  |  |
| Response | Core Team |  |  |  |
| **Response** | **Core Team** |  |  |  |
|  |  |  |  |  |
| **General** | **General** | **General** | **General** | **1: Selection bias - Assessment** |
| Study design: What type of study design is used? | Methods used for analysis: Which methods are used to control for selection bias and confounding? | Ethical clearance | Study registration | 1 - Mechanism of assignment: was the allocation or identification mechanism able to control for selection bias? |
| 1= Natural experiment: randomised or as-if randomised  2= Natural experiment: regression discontinuity (RD)  3= CBA (non-randomised assignment with treatment and contemporaneous comparison group, baseline and end line data collection) – individual repeated measurement  4= CBA pseudo panel (repeated measurement for groups but different individuals)  5= Interrupted time series (with or without contemporaneous control group)  6= Panel data, but no baseline (pre-test)  7 = Comparison group with end line data only | 1 = Statistical matching (PSM, CEM, covariate matching) 2 = Difference in differences (DID) estimation methods 3 = IV-regression (2-stage least squares or bivariate probit) 4 = Heckman selection model 5 = Fixed effects regression 6 = Covariate adjusted estimation 7 = Propensity weighted regression 8 = Comparison of means 9 = Other (please state) | Open answer | Open answer | 1= Yes, 2 = Probably Yes, 3 = Probably No, 4 = No, 8 = Unclear |
|  | - | Provide any details of ethical research clearances granted. Report unclear if this information is not available. | Provide any details of study registration, including registry IDs, etc. |  |

|  |  |  |  |  |
| --- | --- | --- | --- | --- |
| **1: Selection bias - Justification** | **1: Selection bias - Justification** | **2: Confounding - Assessment** | **2: Confounding - Justification** | **2: Confounding - Justification** |
| For regression discontinuity designs | For assignment based non-randomised programme placement and self-selection (studies using a matching strategy or regression analysis, excluding IV) | 2 - Group equivalence: was the method of analysis executed adequately to ensure comparability of groups throughout the study and prevent confounding? | For regression discontinuity design | For non-randomised trials using difference-in-differences methods of analysis |
| Open answer | Open answer | 1= Yes, 2 = Probably Yes, 3 = Probably No, 4 = No, 8 = Unclear | Open answer | Open answer |
| a) Allocation is made based on a pre-determined discontinuity on a continuous variable (regression discontinuity design) and blinded to participants or;     b) if not blinded, individuals reasonably cannot affect the assignment variable in response to knowledge of the participation decision rule;    c) and the sample size immediately at both sides of the cut-off point is sufficiently large to equate groups on average. | a) Participants and non-participants are either matched based on all relevant characteristics explaining participation and outcomes, or;    b) all relevant characteristics are accounted for.**    c) and the data set used contains relevant variable that are measured in a relevant way (i.e. they were not collected for a different purpose initially and therefore are good proxy for some characteristics).    **Accounting for and matching on all relevant characteristics is usually only feasible when the programme allocation rule is known and there are no errors of targeting. It is unlikely that studies not based on randomisation or regression discontinuity can score “YES” on this criterion. There are different ways in which covariates can be taken into account. Differences across groups in observable characteristics can be taken into account as covariates in the framework of a regression analysis or can be assessed by testing equality of means between groups. Differences in unobservable characteristics can be taken into account through the use of instrumental variables (see also question 1.d) or proxy variables in the framework of a regression analysis, or using a fixed effects or difference-in-differences model if the only characteristics which are unobserved are time-invariant |  | a) The interval for selection of treatment and control group is reasonably small OR authors have weighted the matches on their distance to the cut-off point;  b) and the mean of the covariates of the individuals immediately at both sides of the cut-off point (selected sample of participants and non-participants) are overall not statistically different based on t-test or ANOVA for equality of means; c) Significant differences in covariates of the individuals have been controlled in multivariate analysis; and for cluster-assignment, authors control for external cluster-level factors that might confound the impact of the programme. | a) The authors use a difference-in-differences (or fixed effects) multivariate estimation method; b) the authors control for a comprehensive set of individual time-varying characteristics, and for cluster-assignment, authors control for external cluster-level factors that might confound the impact of the programme**; c) and the attrition rate is sufficiently low and similar in treatment and control, or the study assesses that drop-outs are random draws from the sample (for example, by examining correlation with determinants of outcomes, in both treatment and comparison groups);     **Knowing allocation rules for the programme – or even whether the non-participants were individuals that refused to participate in the programme, as opposed to individuals that were not given the opportunity to participate in the programme – can help in the assessment of whether the covariates accounted for in the regression capture all the relevant characteristics that explain differences between treatment and comparison |
| Score “Yes” if criteria a), b), c) are all satisfied   Score "Probably Yes" if there are minor differences in between both sides of the cut-off point but authors convincingly argue that the differences are unlikely to affect the outcome, OR individuals are not blinded and there are low risk of them affecting the assignment but the authors do not mention it.   Score “Unclear” if it is unclear whether participants can affect it in response to knowledge of the allocation mechanism.   Score "Probably No" if there are differences between individuals on both sides of the cut-off point, and there are doubts that the differences are due to individuals altering the assignment OR the participants are blinded but there is evidence that the decisions that determined the discontinuity is based on differences between the two groups or differences in time.   Score “No” if the sample size is not sufficient OR there is evidence that participants altered the assignment variable prior to assignment. If the research has serious concerns with the validity of the assignment process or the group equivalence completely fails, we recommend assessing risk of bias of the study using the relevant questions for the appropriate methods of analysis (cross-sectional regressions, difference-in-difference, etc.) rather than the RDDs questions. | Score “Yes” if a) or b) and c) are satisfied    Score "Probably yes" if a) or b) are addressed for but there is some doubt related to c), OR authors combined statistical matching and difference-in-difference to cope with unobservable differences, OR they only did statistical matching and there was clear rules for selection into the program (no self-selection).    Score “Unclear” if · it is not clear whether all relevant characteristics (only relevant time varying characteristics in the case of panel data regressions) are controlled.     Score "Probably no" if only a statistical matching was done and there was self-selection into the program.    Score “No” if relevant characteristics are omitted from the analysis. |  | Score "Yes, if criterion a), b), c) and d) are addressed.    Score "Probably yes" if b) is not addressed but c) is addressed and differences in means are not large.    Score “Unclear” if insufficient details are provided on controls; or if insufficient details are provided on cluster controls.    Score "Probably no" if b) is not addressed (absence of a difference test or balance table) and there are doubt regarding the continuity on both sides of the cut-off point (a).    Score “No” otherwise. | Score "Yes, if a, b, c, d (if relevant) are addressed and baseline imbalances between groups were relatively low OR the method was combined by a statistical matching.   Score "Probably yes" if all possible variables are controlled for and the selection into the program was done according to clear rules, but baseline imbalances between groups were very large.   Score “Unclear” if insufficient details are provided; or if insufficient details are provided on cluster controls.    Score "Probably no" if some time-varying characteristics are not controlled for and the program was self-selected by the intervention groups.   Score “No” if any of the criterion is not addressed. |

|  |  |  |  |  |
| --- | --- | --- | --- | --- |
| **2: Confounding - Justification** | **2: Confounding - Justification** | **3: Performance bias - Assessment** | **3: Performance bias - Justification** | **4: Spill-overs, cross-overs and contamination - Assessment** |
| For statistical matching studies including propensity scores (PSM) and covariate matching**  **Matching strategies are sometimes complemented with difference-in-difference regression estimation methods. This combination approach is superior since it only uses in the estimation the common support region of the sample size, reducing the likelihood of existence of time-variant unobservable differences across groups affecting outcome of interest and removing biases arising from time-invariant unobservable characteristics. | For regression-based studies using cross sectional data (excluding IV) | 3 - Performance bias: was the process of being observed free from motivation bias? | Performance bias - Justification | 4 - Spill-overs, cross-overs and contamination: was the study adequately protected against spill-overs, cross-overs and contamination? |
| Open answer | Open answer | 1= Yes, 2 = Probably Yes, 3 = Probably No, 4 = No, 8 = Unclear | Open answer | 1= Yes, 2 = Probably Yes, 3 = Probably No, 4 = No, 8 = Unclear |
| a) Matching is either on baseline characteristics or time-invariant characteristics which cannot be affected by participation in the programme; and the variables used to match are relevant (for example, demographic and socio-economic factors) to explain both participation and the outcome (so that there can be no evident differences across groups in variables that might explain outcomes); and, for cluster-assignment, authors control for external cluster-level factors that might confound the impact of the programme b) in addition, for PSM Rosenbaum’s test suggests the results are not sensitive to the existence of hidden bias;  c) and, with the exception of Kernel matching, the means of the individual covariates are equated for treatment and comparison groups after matching;  d) different matching methods including varying sample sizes yields the same results and authors take into account the use of control observations multiple times against the same treatment in their standard error calculation. | a) The study controls for relevant confounders that may be correlated with both participation and explain outcomes (for example, demographic and socio-economic factors at individual and community level) using multivariate methods with appropriate proxies for unobservable covariates, and, for cluster-assignment, authors control particularly for external cluster-level factors that might confound the impact of the programme; b) and a Hausman test with an appropriate instrument suggests there is no evidence of endogeneity**; c) and none of the covariate controls can be affected by participation; d) and either, only those observations in the region of common support for participants and non-participants in terms of covariates are used, or the distributions of covariates are balanced for the entire sample population across groups;    **The Hausman test explores endogeneity in the framework of regression by comparing whether the OLS  and the IV approaches yield significantly different estimations. However, it plays a different role in the  different methods of analysis. While in the OLS regression framework the Hausman test mainly explores  endogeneity and therefore is related with the validity of the method, in IV approaches it explores whether the  author has chosen the best available strategy for addressing causal attribution (since in the absence of  endogeneity OLS yields more precise estimators) and therefore is more related with analysis reporting bias. | a) For data collected in the context of a particular intervention trial (randomised or non-randomised assignment), the authors state explicitly that the process of monitoring the intervention and outcome measurement is blinded, or argue convincingly why it is not likely that being monitored could affect the performance of participants in treatment and comparison groups in different ways (such as resulting in Hawthorne or John Henry effects).    b) The study is based on data collected in the context of a survey, and not associated with a particular intervention trial, or data are collected from administrative records or in the context of a retrospective (ex post) evaluation. | Justification for coding decision  (Include a brief summary of justification for rating, mentioning your response to all sub questions, cite relevant pages). | a) There was no implementation issues that might have led the control participants to receive the treatment (implementer's mistake).  b) The intervention is unlikely to spill-over to comparisons (e.g. participants and non-participants are geographically and/or socially separated from one another and general equilibrium effects are not likely) or the potential effects of spill overs were measured (e.g. variation in the % of unit within a cluster receiving the treatment).  c) There is no risk of contamination by external programs: the treatment and comparisons are isolated from other interventions which might explain changes in outcomes.   d) There is nothing in the surveys that might have given the control participants an idea of what the other group might receive OR they did but there is no risk that this has changed their behaviours; AND the survey process did not reveal information to the control group that they did not have before (e.g. the study aims to measure increase in take up of a service or product that participants might not know about)  Authors might put something in place in the design of the study that allows to control for that survey effect (e.g. a pure control with no monitoring except baseline end line) |
| Score "Yes, if a, b, c, and d (if relevant) are addressed.    Score "Probably yes" if the selection into the program was done according to clear rules, which are used for the matching but there are slight imbalances remaining after matching.    Score “Unclear” if relevant variables are not included in the matching equation, or if matching is based on characteristics collected at end line; or if insufficient details are provided on cluster controls.    Score "Probably no" if the program was self-selected by the intervention groups or participants OR if the selection into the program was done according to clear rules but there is no baseline data available to match the participants or groups on.    Score “No” if matching was done based on variables that are likely to be affected by the program or any other scenario that affect a), b) c) or d). | Score "Yes, if a, b, c and d are addressed.    Score "Probably yes" if all criterion are addressed but authors did not report the Hausman test (b).    Score “Unclear” if relevant confounders are controlled but appropriate proxy variables or statistical tests are not reported; or if insufficient details are provided on cluster controls.     Score "Probably no" if any of the criterion other than b) is not addressed.    Score “No" if none of the criterion are addressed. | Score “Yes” if either criterion a) or b) are satisfied;    Score "Probably yes" if the study is based on survey data collected during a trial and there is no obvious issue with the monitoring processes but authors do not mention potential risks.    Score “Unclear” if it is not clear whether the authors use an appropriate method to prevent Hawthorne and John Henry Effects (e.g. blinding of outcomes and, or enumerators, other methods to ensure consistent monitoring across groups). Hawthorne effects may result where participants know that they are being observed and John Henry Effects may result from participant knowledge of being compared.    Score "Probably no" if there was imbalance in the frequency of monitoring in intervention groups, which might have influenced participants' behaviours.    Score "No" eif neither criterion a) or b) are satisfied; |  | Score “Yes” if criterion a), b), c) and d) are satisfied;    Score "Probably yes" if there is no obvious problem but there is no information reported on potential risks related to spill overs, contamination, or survey effects in the control group OR if there were issues with spill-overs but they were controlled for or measured.    Score “Unclear” if spill-overs, cross-overs, survey effects and/or contamination are not addressed clearly.    Score "Probably no" if any of the criterion a), b), c) or d) are not satisfied but the scale of the issue is not clear.    Score “No” if any of the criterion a), b), c) or d) are not satisfied and happened at a large scale in the study. |

|  |  |  |  |  |
| --- | --- | --- | --- | --- |
| **4: Spill-overs, cross-overs and contamination - Justification** | **5: Outcome measurement bias - Assessment** | **6: Reporting bias - Assessment** | **6: Reporting bias - Justification** | **7: Other bias - Assessment** |
| Spill-overs, cross-overs and contamination - Justification | 5 - Outcome measurement bias | 6 - Selective analysis reporting: was the study free from selective analysis reporting? | Analysis reporting bias - Justification | 7 - Other risks of bias: Is the study free from other sources of bias? |
| Open answer | 1= Yes, 2 = Probably Yes, 3 = Probably No, 4 = No, 8 = Unclear | 1= Yes, 2 = Probably Yes, 3 = Probably No, 4 = No, 8 = Unclear | Open answer | 1= Yes, 4 = No |
| Justification for coding decision  (Include a brief summary of justification for rating, mentioning your response to all sub questions, cite relevant pages). | a) Outcome assessors are blinded or the outcome measures are not likely to be biased by their judgement.   b) For self-reported outcomes: respondents in the intervention group are not more likely to have accurate answers due to recall bias;  c) For self-reported outcomes: respondents do not have incentives to over/under report something related to their performance or actions, OR researchers put in place mechanisms to reduce the risk of reporting bias (researchers not strongly involved in the implementation of the program and it is clear that their answers to the survey will not affect what they receive in the future) OR authors have measured the risks of bias through falsification tests or measuring the effect on placebo outcomes in cases where there was a risk of reporting bias.  d) Timing issue: the data collection period did not differ between intervention and comparison group, the baseline data is not likely to be affected by the beginning of the intervention or affects a small percentage of the study participants. | a) a pre-analysis plan is published, especially for prospective NRS but it should also be for retrospective studies b) authors use ‘common’ methods of estimation (i.e. credible analysis method to deal with attribution given the data available) ; c) There is no evidence that outcomes were selectively reported (e.g. results for all relevant outcomes in the methods section are reported in the results section) ; d) Requirements for specific methods of analysis:  - For PSM and covariate matching: (a) Where over 10% of participants fail to be matched, sensitivity analysis is used to re-estimate results using different matching methods (Kernel Matching techniques); (b) For matching with replacement, no single observation in the control group is matched with a large number of observations in the treatment group.  - For IV (including Heckman) models, (a) The authors test and report the results of a Hausman test for exogeneity (p≤0.05 is required to reject the null hypothesis of exogeneity); (b) the coefficient of the selectivity correction term (Rho) is significantly different from zero (P<0.05) (Heckman approach).   - For studies using multivariate regression analysis, authors conduct appropriate specification tests (e.g. testing robustness of results to the inclusion of additional variables, or (very rare) reporting results of multicollinearity test etc). | Justification for coding decision  (Include a brief summary of justification for rating, mentioning your response to all sub questions, cite relevant pages). | Score “Yes” if the reported results do not suggest any other sources of bias.  Score “No” if other potential threats to validity are present, and note these here (e.g. coherence of results, survey instruments used are not reported) |
|  | Score “Yes” if criterion a), b), c) and d) are satisfied:    Score "Probably yes" if there is a small risk related to any of a), b), c) or d) and there is no more information provided to justify the absence of bias OR if there was a high risk of bias but authors have either controlled it in their design or measured it with a placebo outcomes.    Score “Unclear” if it there is a high risk related to any of a), b), c) or d) and there is no more information provided to justify the absence of bias.     Score "Probably no" if there are high risk related to a), b), c) or d) and it is clear that authors were not able to control for this bias.    Score “No” if there is evidence of bias. | Score “Yes” if a), b), c) and d) are satisfied OR if a) is not met and it is a retrospective NRS. Score "Probably Yes" if authors combined methods and reported relevant tests (d) only for one method OR if all the criteria are met except for a) and it is a prospective NRS Score "Unclear" if intended outcomes not specified in the paper OR if any of the requirements for d) are not reported.  Score "Probably No" if b) is addressed, but authors did not present results for all outcomes announced in the method section OR did not meet requirement d) although reported.  Score “No” if authors use uncommon or less rigorous estimation methods such as failure to conduct multivariate analysis for outcomes equations OR if some important outcomes are subsequently omitted from the results or the significance and magnitude of important outcomes was not assessed. |  |  |

| **7: Other bias - Justification** | **8: External validity** |
| --- | --- |
| Other risks of bias - Justification | 8 - External validity |
| Open answer | Open answer |
| Justification for coding decision  (Include a brief summary of justification for rating, mentioning your response to all sub questions, cite relevant pages). | Open answer- what do authors say about external validity, if anything? |

# **Additional References**

Grames, E. M., Stillman, A. N., Tingley, M. W., & Elphick, C. S. (2019). An automated approach to identifying search terms for systematic reviews using keyword co-occurrence networks. *Methods in Ecology and Evolution*, *10*(10), 1645–1654.

Wooldridge, J. M. (2021). *Two-Way Fixed Effects, the Two-Way Mundlak Regression, and Difference-in-Differences Estimators*. Rochester, NY: Social Science Research Network. Available at: https://www.ssrn.com/abstract=3906345

1. The supplementary material in the final report will provide details of any adaptations of the search string required for other websites and databases. [↑](#footnote-ref-2)
2. These search filters were taken from Cochrane EPOC LMIC (<https://epoc.cochrane.org/lmic-filters>). [↑](#footnote-ref-3)
3. These search filters were taken from Cochrane EPOC LMIC (<https://epoc.cochrane.org/lmic-filters>). [↑](#footnote-ref-4)
